# Biocatalytic Conversion of Carrageenans for the Production of 3,6-Anhydro-D-galactose

**DOI:** 10.1021/acs.jafc.3c08613

**Published:** 2024-03-05

**Authors:** Alexander Fuchs, Dennis Romeis, Enrico Hupfeld, Volker Sieber

**Affiliations:** † Chair of Chemistry of Biogenic Resources, TUM Campus Straubing for Biotechnology and Sustainability, Technical University of Munich, Schulgasse 16, 94315 Straubing, Germany; ‡ SynBioFoundry@TUM, Technical University of Munich, Schulgasse 22, 94315 Straubing, Germany; § Catalytic Research Center, Ernst-Otto-Fischer-Straße1, 85748 Garching, Germany; ∥ School of Chemistry and Molecular Biosciences, The University of Queensland, 68 Copper Road, St. Lucia 4072, Australia

**Keywords:** blue biotechnology, carrageenan, sulfatase, biotransformation, feedstock utilization, red algae

## Abstract

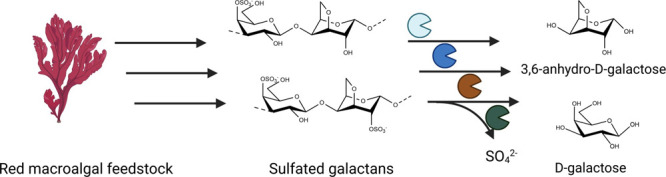

Marine biomass stands out as a sustainable resource for generating value-added chemicals. In particular, anhydrosugars derived from carrageenans exhibit a variety of biological functions, rendering them highly promising for utilization and cascading in food, cosmetic, and biotechnological applications. However, the limitation of available sulfatases to break down the complex sulfation patterns of carrageenans poses a significant limitation for the sustainable production of valuable bioproducts from red algae. In this study, we screened several carrageenolytic polysaccharide utilization loci for novel sulfatase activities to assist the efficient conversion of a variety of sulfated galactans into the target product 3,6-anhydro-D-galactose. Inspired by the carrageenolytic pathways in marine heterotrophic bacteria, we systematically combined these novel sulfatases with other carrageenolytic enzymes, facilitating the development of the first enzymatic one-pot biotransformation of ι- and κ-carrageenan to 3,6-anhdyro-D-galactose. We further showed the applicability of this enzymatic bioconversion to a broad series of hybrid carrageenans, rendering this process a promising and sustainable approach for the production of value-added biomolecules from red-algal feedstocks.

## 1 Introduction

**Figure 1:**
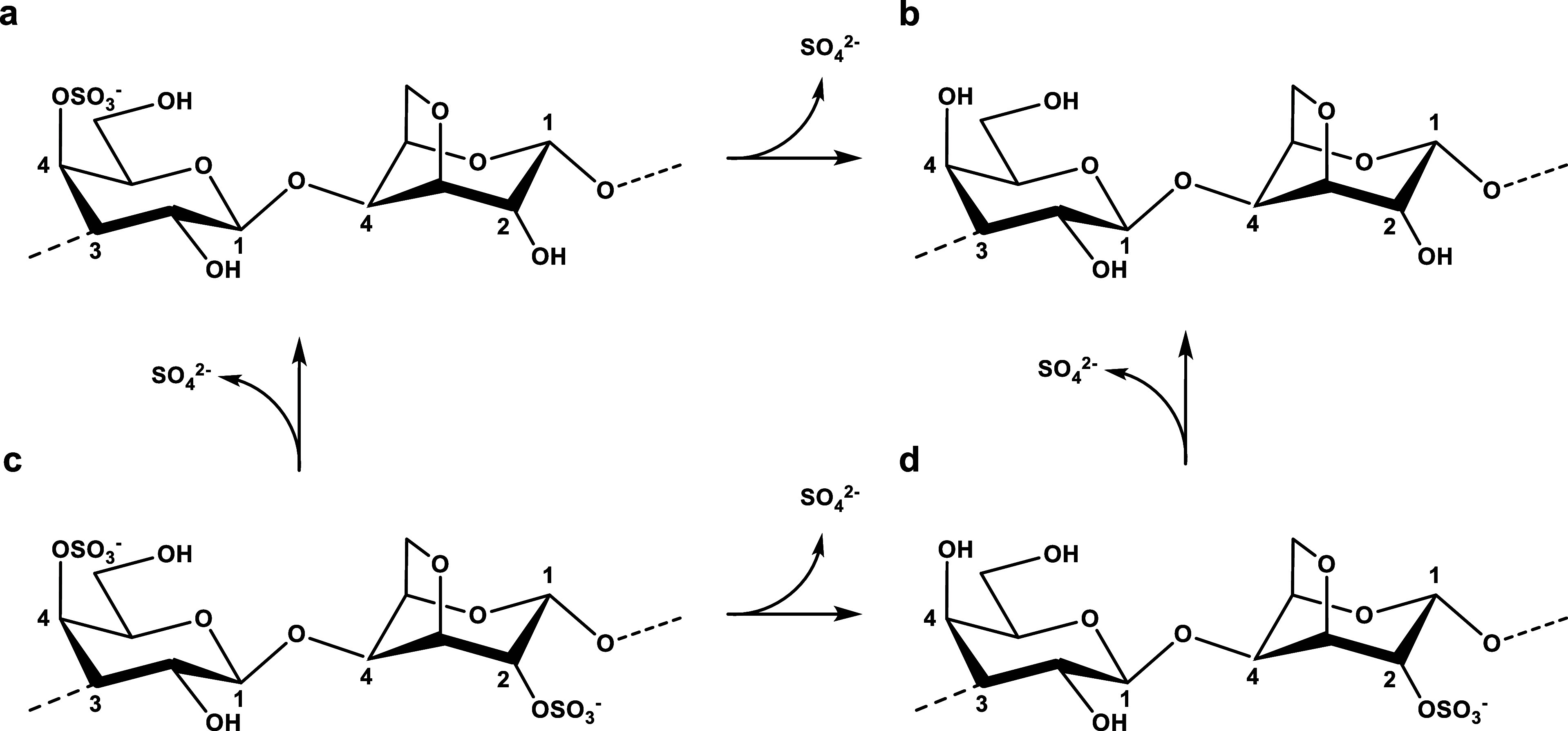
Chemical structures of the idealized repeating units of (a) κ-(G4S-DA), (b) β-(G-DA), (c) ι-(G4S-DA2S) and (d) α-(G-DA2S)-carrageenan. The arrows between the polymer structures illustrate possible reactions catalyzed by sulfatases.

Carrageenans, a family of sulfated polysaccharides derived from red algae, have attracted considerable scientific and industrial interest due to their unique structural complexity and multifunctional properties. These abundant and renewable biopolymers are widely used in various industries such as food, pharmaceuticals, cosmetics, and biotechnology.^1^ The linear backbone of carrageenans generally consists of alternating β-1,4- and α-1,3-linked D-galactose (D-gal) derivatives.^2^ A special feature of carrageenans is the presence of several galactose derivatives, which are generally a unique 3,6-anhydro-ring (DA) at the 4-linked residue exclusively occurring in this polymer, as well as the decoration with sulfate groups or, in rare cases, methyl esters, pyruvic acid ketals or even monosaccharides like galactose or β-D-xylopyranose.^3^ These polymers are organized in disaccharide repeating units of sulfated D-gal and DA, called carrabioses, and are classified according to their substitutions on the different hydroxyl groups. In nature, the mentioned substitutions lead to enormous structural diversity and complexity of carrageenans that is reflected in the classification of at least 10 different idealized types, with κ-carrageenan, ι-carrageenan, and λ-carrageenan being the most common and commercially used forms.^1^ In an ideal structural homogeneity, κ-carrageenan is sulfated at position 4 on D-gal (G4S) and lacks sulfation on DA, while in ι-carrageenan, DA is additionally sulfated at position 2 (DA2S) (Figure 1).

A promising tool to tune the sulfation patterns of sulfated polysaccharides has strongly emerged during the past decade with the identification and classification of new polysaccharide-active sulfatases from marine microorganisms, which emphasizes microbes from these habitats as a valuable resource for new biocatalysts.^4−8^ These enzymes represent a central key step in the metabolic cascading of sulfated polysaccharides to their monosaccharides and are therefore of critical relevance to the biotechnological utilization and upgrading of these polymers.^9−11^ Sulfatases are commonly classified into four different families, with the S1-family representing the largest of them and consisting of more than 100 subfamilies.^5^ Members of this family belong to the category of formylglycine-dependent (fGly-dependent) sulfatases since either a cysteine (Cys) or serine (Ser) residue in the active side undergoes a post-translational modification to a catalytically active formylglycine residue.^10^ Up to now, most polysaccharide active sulfatases, capable of working on an exo- or endomode of action, belong to this class of enzymes, which proves it the most promising sulfatase family for polysaccharide processing. Within this handful of sulfatases, few were found to be active on carrageenans, and many of them belong to subclass S1_19. These carrageenan sulfatases (CgS) are almost exclusively active on the G4S of the polymers or their derived oligosaccharides to yield α- or β-carrabiose units from ι- or κ-carrageenans, respectively, while only one S1_17 sulfatase from *Zobellia galactanivorans* Dsij^T^ and an S1_81 sulfatase from *Pseudoalteromonas atlantica* T6c could remove sulfate from DA2S at the nonreducing end of α-carrageenan oligosaccharides.^11,12^ However, an enzymatic activity enabling the direct desulfation from ι- to κ-neocarrabioses has not been identified so far. Generally, these polysaccharide-active sulfatases are localized within carbohydrate-specific gene clusters that are coresponsible for the metabolism of these polymers, so-called polysaccharide utilization clusters (PULs), which could therefore be a possible starting point for the screening of sulfatases with previously unidentified activities.^11,13,14^

In addition to their important polymer properties, carrageenan oligosaccharides are known to exhibit antioxidant, antitumor, antiviral, and antibacterial effects, while the carrageenan-exclusive rare sugar DA has been reported to have anti-inflammatory and skin-whitening effects in vitro and could be used as a novel anticariogenic sugar substitute to prevent dental caries.^15−17^^18−20^^21^ Further studies have shown that 3,6-anhydro-L-galactose, the stereoisomer of DA that is present in agarose, can be converted into isosorbide, an important platform chemical for the production of biopolymers, highlighting the great potential of this little-studied sugar for biotechnological and chemical purposes.^22^ κ-carrageenans usually show a DA content varying between 28 and 35%, whereas in ι -carrageenan DA constitutes between 25 and 30% of the polymer weight.^23^

However, despite its promising potential, its natural uniqueness, and its source from a renewable feedstock, the availability of studies dealing with the production of DA from carrageenans remains poor. In contrast, approaches to the production of 3,6-anhydro-L-galactose from agarose are more prominent, presumably due to the much less complex structure of agarose, which does not require the action of sulfatases for its utilization.^24,25^

In recent studies, DA has been produced using κ-carrageenan with acid-catalyzed hydrolysis, but this is associated with a nonspecific degree of polymerization of the oligosaccharides produced, unnecessary chemical modifications, and undesirable chemical byproducts, as well as degradation of DA.^26^

Recently, the first purely enzymatic approach for the production of DA from κ-carrageenan was presented.^27^ By combining a recombinant κ-carrageenase and the cell-free extract of the carrageenolytic heterotrophic bacterium *Colwellia echini* A3^T^ in three consecutive steps with intermediate enzyme denaturation, enzymatic production of DA was demonstrated, but the desulfation step in the process still remained a “black box”. This further highlights the need for more detailed studies to identify carrageenan sulfatases for suitable biotransformations.

However, despite, or because of, the great structural diversity of carrageenans, there have been no reports on the production of DA from sulfated galactans other than κ-carrageenan, which could be due to the limited availability of sulfatases capable of effectively removing the 3,6-anhydro-α-D-galactose-2-sulfate (DA2S) residue.

To address this issue, we present here, for the first time, a cell-free enzymatic pathway for the production of DA from different sulfated galactans, thereby utilizing the action of a novel exo-DA2S-sulfatase activity. The presented process is constructed based on recently explored or speculated pathways for carrageenan degradation in marine heterotrophic bacteria matching identified and putative carrageenolytic enzymes from different PULs.

## 2 Materials and Methods

### 2.1 Materials

All chemicals and commercial enzymes were purchased in high purity from Roth, Sigma-Aldrich, VWR, Carbosynth, and Fisher Scientific unless otherwise stated. 3,6-Anhydro-D-galactose was purchased from Dextra Laboratories (Reading, UK). *Escherichia coli* (*E. coli*) NEB Turbo cells (NEB, USA) were used as cloning strains. *E. coli* BL21(DE3) (NEB, USA) was used for heterologous gene expression.

Commercial κ-carrageenan and ι-carrageenan were purchased from Carl Roth (Karlsruhe, Germany). Furcellaran was purchased from Carbosynth (Compton, UK). ι/ν-carrageenan was extracted from *Eucheuma spinosum*, κ/μ-carrageenan from *Κappaphycus alvarezii*, and κ/ι-hybrid carrageenan from gametophytes of *Chondrus crispus*. *Chondrus Crispus*, *Kappaphycus alvarezii*, and *Kappaphycus striatus* biomass was a friendly gift from Alan T. Critchley (Verschuren Centre for Sustainability in Energy and Environment, Sydney, Nova Scotia, Canada), Anicia Q. Hurtado (Integrated Services for the Development of Aquaculture and Fisheries (ISDA) Inc., Jaro, Philippines) and Shienna Mae C. Gonzaga (The Marine Science Institute, Quezon City, Philippines), and *Eucheuma spinosum* biomass was provided by by Flower Msuya, Zanzibar Seaweed Cluster (Zanzibar, Tanzania).

### 2.2 Cloning, Expression, and Enzyme Purification

The genomic DNA of *Cellulophaga algicola* DSM 14327, *Cellulophaga baltica* DSM 24729, *Cellulophaga lytica* DSM 7489, *Saccharicrinis fermentans* DSM 9555, *Echinicola pacifica* DSM 19836 and *Weizmannia coagulans* DSM 2356 was purchased from DSMZ (Braunschweig, Germany). *Pseudoalteromonas atlantica* T6c genomic DNA was purchased from ATCC (Manassas, USA). *Pseudoalteromonas carrageenovora* ATCC 43555^T^ genomic DNA was extracted from the strain purchased from DSMZ. Cultivation was performed according to the DSMZ protocol, and isolation of gDNA was performed using the DNeasy UltraClean Microbial Kit (Qiagen, Germany). Genes in this work were PCR-amplified from genomic DNA removing putative signal peptides predicted using SignalP 5.0.^28^ Codon-optimized genes were ordered from GENEART AG (Regensburg, Germany). The genes were cloned into the pET28a vector (Invitrogen, Germany) to provide N-terminal histidine tags. Where possible, genes were cloned into a modified pET28a vector using a golden gate approach.^29^ WcBGH was cloned in the pACYC vector. The primers are summarized in Table S1. The pBAD/myc-his A Rv0712 (FGE) was a gift from Carolyn Bertozzi.^30^

Recombinant proteins were expressed in *E. coli* (BL21) DE3. For expression, an overnight culture was transferred to terrific broth media containing 50 μg mL^–1^ kanamycin for pET28-vector constructs, or 25 μg mL^–1^ chloramphenicol for pACYC vector constructs, and grown to an OD_600_ of 0.6 at 37 °C, at which time the temperature was lowered to 18 °C and 1 mM IPTG was added. For sulfatase expression, cells were cotransformed with the constructs and the pBAD/myc-his A Rv0712 vector and grown in 50 μg mL^–1^ kanamycin sulfate and 100 μg mL^–1^ ampicillin. Cells were grown at 37 °C until the OD_600_ reached 0.5, then the temperature was lowered to 18 °C, and FGE expression was induced with 0.02% (w/v) l-arabinose. After 90 min, sulfatase expression was induced with 1 mM IPTG, and the expression was continued at 18 °C for 20 h. The cells were harvested at 5000 × *g* for 10 min and the pellets were resuspended in buffer A (20 mM Tris-HCl, 300 mM NaCl, 20 mM imidazole, pH 8.0) containing a total of 5 μg/mL DNase and 2 mM MgCl_2_. Cells were then disrupted by sonication (80% and cycle 0.5 s) in an ice bath for 20 min, and the lysate was cleared by centrifugation at 35,000 × *g* for 30 min. The cell-free supernatant was filtered through a 0.45 μm cellulose filter (VWR, Germany) before application to a kta pure FPLC system (Cytiva, Germany). His-tagged enzymes were purified using a 5 mL HisTrap FF Crude (Cytiva, Germany) at a flow rate of 5 mL min^–1^ and an elution buffer (20 mM Tris-HCl, 300 mM NaCl, 500 mM imidazole, pH 8.0) was used to elute the His-tagged proteins from the column. After purification, the buffer was exchanged with 20 mM Tris-HCl, pH 7.5, for all proteins using a HiPrep 26/10 desalting column (Cytiva, Germany). After buffer exchange, all enzymes were snap-frozen in liquid nitrogen and stored at −80 °C until further use. Enzyme purity and size were analyzed by SDS-PAGE. Protein concentration was determined using a NanoPhotometer P-Class (IMPLEN, Germany). Absorbance was measured at 280 nm, and concentration was determined by applying the molecular weight and extinction coefficients of the respective enzymes.

### 2.3 Extraction of Carrageenans from Red Algal Biomass

Extraction of carrageenans from red algal raw materials was performed using 2 g of dried biomass in a volume of 100 mL. For alkaline pretreatment, the raw material was soaked in 4% (w/v) KOH for 1 h and treated at 90 °C for 2 h with stirring. To ensure the presence of μ- and ν-moieties in the final product, the alkaline pretreatment was omitted. The suspension was cooled to room temperature, and the biomass was washed and resuspended in distilled water (pH 7–8). Carrageenan extraction was then carried out at 90 °C for 2 h. The viscous suspensions were removed from the residual algal biomass by centrifugation (50,000 × *g*, 20 min, 40 °C) to remove insoluble compounds. The clear supernatant was then precipitated in two volumes of 2-propanol, and the precipitated polymer was dried in a VDL53 vacuum oven (Binder, Germany) at 40 °C for 24 h. The dried carrageenans were weighed to determine the extraction yield and stored at RT.

### 2.4 Preparation of Carrageenan Oligosaccharides

Carrageenan oligosaccharides were prepared by extensive hydrolysis of 0.5% (w/v) carrageenans in 50 mL of Tris-HCl pH 7.5 at 37 °C for 48 h applying 2 mg of BovGH16. After hydrolysis, the enzyme was inactivated at 95 °C for 10 min and centrifuged for 20 min at 30,000*g*, and the supernatant was lyophilized. Dried oligosaccharides were stored at −20 °C.

### 2.5 Setup of the Carrageenolytic Cascade

Carrageenans were prepared in a 1% (w/v) solution in 20 mM Tris-HCl pH 7.5. All reactions were performed in a volume of 100 μL in 1.5 mL Eppendorf tubes. The initial enzyme mix containing 0.0075 mg mL^–1^ BovGH16, 2.5 mg mL^–1^ CaCgS2, 0.667 mg mL^–1^ EpCgS2, 0.75 mg mL^–1^ CaGH127_1 and 0.166 mg mL^–1^ WcBGH was prepared in the tube to a volume of 50 μL and the reaction was started by the addition of 50 μL of substrate. Reactions were performed in triplicates at 37 °C for 16 h before further analysis.

### 2.6 Determination of Enzyme Characteristics

All spectrophotometric analyses of enzymes in this study were performed in 96 well F-bottom plates in a volume of 200 μL in a Biotek epoch-2 microplate spectrophotometer (Biorad, USA). For all enzymes, pH characterization was generally performed using 20 mM MES (5.5 to 6.5), 20 mM Bis-Tris (6.0 to 7.0), 20 mM MOPS (6.5 to 7.5), 20 mM Tris-HCl (7.0 to 9.0) and 20 mM HEPES (pH 7.0 to 8.5). Influences of salts and other components were determined by dilution of stock solutions in the reaction matrix.

### 2.7 Determination of Sulfatase Activity

Determination of sulfatase activity by analysis of free sulfate in the reaction media was performed by a previously developed sulfate assay.^31^ Initial enzyme characterizations were carried out, if not stated otherwise, at a carrageenan concentration of 0.25% (w/v) in a volume of 100 μL at 37 °C in 20 mM Tris-HCl pH 7.5 and 0.5 mM CaCl_2_ using 50 μg of the enzyme. When necessary, the buffer was exchanged with 10 kDa centrifuge filters (Merck KGaA, Germany). For all reactions, control samples were prepared with the same conditions and the previously heat-inactivated enzyme. For enzyme characterization, the reactions were stopped after 30 min by heat inactivation at 95 °C for 10 min. For assaying the released sulfate, the samples were diluted at least five times in ddH_2_O to ensure an absorbance signal in the calibration range. The relative activity was then defined as the percentage of the activity observed for the conditions yielding the highest sulfate release. All measurements were performed in triplicates.

### 2.8 Determination of β-Galactosidase/exo-(α-1,3)-3,6-anhydro-D-galactosidases Activity

The substrate for assaying exo-(α-1,3)-3,6-anhydro-D-galactosidases activity, 4-nitrophenyl-3,6-anhydro-α-D-galactopyranoside, was synthesized as previously described by Wallace et al.^32^ β-galactosidase (BGH) activity was determined using 4-para-nitrophenol-β-D-galactopyranoside. Standard reactions were performed in 200 μL of 20 mM Tris-HCl pH 7.5 at 37 °C and started by the addition of 20 μL of the enzyme in a suitable dilution. Para-nitrophenol (pNP) release was followed by the measurement at 405 nm, and enzyme activity was calculated based on the extinction coefficient of pNP at pH 7.5. For the determination of pH dependency, the extinction coefficient of pNP at the respective pH was used for calculation.

### 2.9 Determination of Carrageenase Activity

The activity of purified BovGH16 was quantified using the 3,5-dinitrosalicylic acid (DNS) assay for the quantification of reducing sugars using D-gal as the standard. Standard reactions contained 5 g L^–1^ carrageenans in 20 mM Tris-HCl pH 7.5 at 37 °C. For this, 60 μL samples of enzyme reactions were mixed with 120 μL of DNS reagent and incubated for 10 min at 95 °C. 150 μL were transferred to a 96-well microtiter plate and absorption was measured at 540 nm.

### 2.10 Determination of Dehydrogenase Activities

*Cellulophaga algicola* DSM 14237 3,6-anhydro-D-galactose-dehydrogenase (CaDADH) activity was measured directly by the reduction of NAD^+^. Standard reactions contained 1 mM NAD^+^ and 0 to 1 mM of DA for kinetic analysis or 1 mM DA for characterization and were performed in 20 mM Tris-HCl pH 7.5 at 37 °C. Reactions were started by the addition of an enzyme.

### 2.11 Quantitative Analysis of DA

Before quantification, reaction mixtures were diluted 1:5 or 1:10 in 2.5 mM H_2_SO_4_ and filtered through 10 kDa centrifugal filters (Merck, Germany).

DA was analyzed and quantified using an HPLC (Ultimate300 HPLC-system, Dionex Softron GmbH, Germany) system coupled to a UV-detector at 210 nm and an RI detector, equipped with a Rezex ROA-Organic Acid H+ (8%) 300 × 7.8 mm LC Column (Phenomenex, Germany). Separation was conducted in an isocratic run with 2.5 mM H_2_SO_4_ at 70 °C for 35 min. Signal analysis and amount calculation were conducted by using Chromeleon (Thermo Fisher Scientific, USA).

Alternatively, a spectrophotometric quantitative analysis of DA by an enzymatic assay based on the activity of CaDADH was performed. In total, 10 μg of the pure recombinant enzyme (14.1 U mg^–1^) was incubated in 20 mM Tris-HCl (pH 7.5) containing the reaction mixture and 1 mM NAD^+^ at 37 °C. Absorbance was followed for 30 min at 340 nm until it reached a plateau, and the concentration of NADH/H^+^, which is equimolar to produced DA, was calculated (molar extinction coefficient = 6.22 mM^–1^ cm^–1^). Assays were performed in triplicate, and the reaction mixture was added in a suitable dilution (1:5 to 1:10).

### 2.12 Melting Point Analysis

Melting point analysis was performed in 25 μL reaction volume using 2 μL of SYPRO Orange (1:80 dilution), 2 μL of protein (1 mg mL^–1^), and 20 mM Tris-HCl pH 7.5. Measurements were performed using a CFX96 Touch Real-Time PCR detection system (Biorad, USA), and the temperature was increased in increments of 1 °C from 4 to 100 °C with 1 min per kelvin increase. The melting curves were prepared as described in the manufacturer‘s instructions. Melting point data were derived from the minimum of the negative derivative of the fluorescence curve versus temperature.

### 2.13 Nuclear Magnetic Resonance (NMR)

To determine the substrate specificity and regioselectivity of sulfatases by ^1^H NMR, 1 mL of polymeric carrageenans (10 mg mL^–1^ in 20 mM Tris-HCl, pH 7.5, 0.5 mM CaCl_2_) were incubated with 2 mg of sulfatases at 37 °C for 24 h. For oligosaccharide active sulfatases, the mixture was coincubated with 10 μg of BovGH16. The reactions were heat-inactivated for 5 min at 95 °C. Polymer samples were precipitated with two volumes of isopropanol and dried in a VDL53 vacuum oven (Binder, Germany) at 40 °C overnight. Oligosaccharide samples were directly concentrated in a vacuum centrifuge and two times exchanged with D_2_O (99.9%). Samples for ^1^H NMR were dissolved in D_2_O to around 10 mg mL^–1^. The samples were transferred to 5 mm NMR tubes and spectra were recorded at 70 °C on a JNM-ECA 400 MHz spectrometer (JEOL, Japan). Chemical shifts were expressed in ppm, and for these experiments, 128 scans were performed. NMR spectra assignation was supported by comparison with literature values for similar compounds.

### 2.14 Phylogenetic Analysis

The set of sulfatases used for the phylogenetic analysis contained all identified sulfatases of families S1_19, S1_7, and S1_81 in this work. The sequence alignment and phylogenetic tree construction were performed using MEGA-X software.^33^ Sequence alignment was performed using the MUSCLE algorithm and the phylogenetic tree was developed using the maximum likelihood algorithm embedded in MEGA-X using default parameters.^34,35^ The sulfatase families were annotated using the SulfAtlas database.^5^

## 3 Results and Discussion

### 3.1 Cell-Free Process for Carrageenan Utilization

The natural pathway of carrageenan degradation was extensively studied for several marine bacteria, including *Zobellia galactanivorans* Dsij^T^, *Pseudoalteromonas fuliginea* PS47, *Paraglaciecola hydrolytica* S66^T^, and *Colwellia echini* A3^T^.^11,14,36,37^ In a common initial step, polymeric ι - and κ-carrageenans are hydrolyzed by carrageenases yielding neocarrageenan oligosaccharides with varying degrees of polymerization. In the further process, depending on the carrageenolytic system, a combination of exo- or endo-DA2S and G4S sulfatases from the S1-family, exo-(α-1,3)-3,6-anhydro-D-galactosidases from the GH127/GH129 class, and β-galactosidases can convert the oligosaccharides to D-gal and DA in a sequential manner. For *Z.galactanivorans* Dsij^T^, it was found that a family S1_17 sulfatase is responsible for the desulfation of DA2S-residues on the nonreducing end of α-carrageenan oligosaccharides to β-carrageenan moieties, allowing the utilization of DA2S-sulfated oligo-carrageenans to the metabolism after they faced pretreatment by a G4S sulfatase.^11^

**Figure 2:**
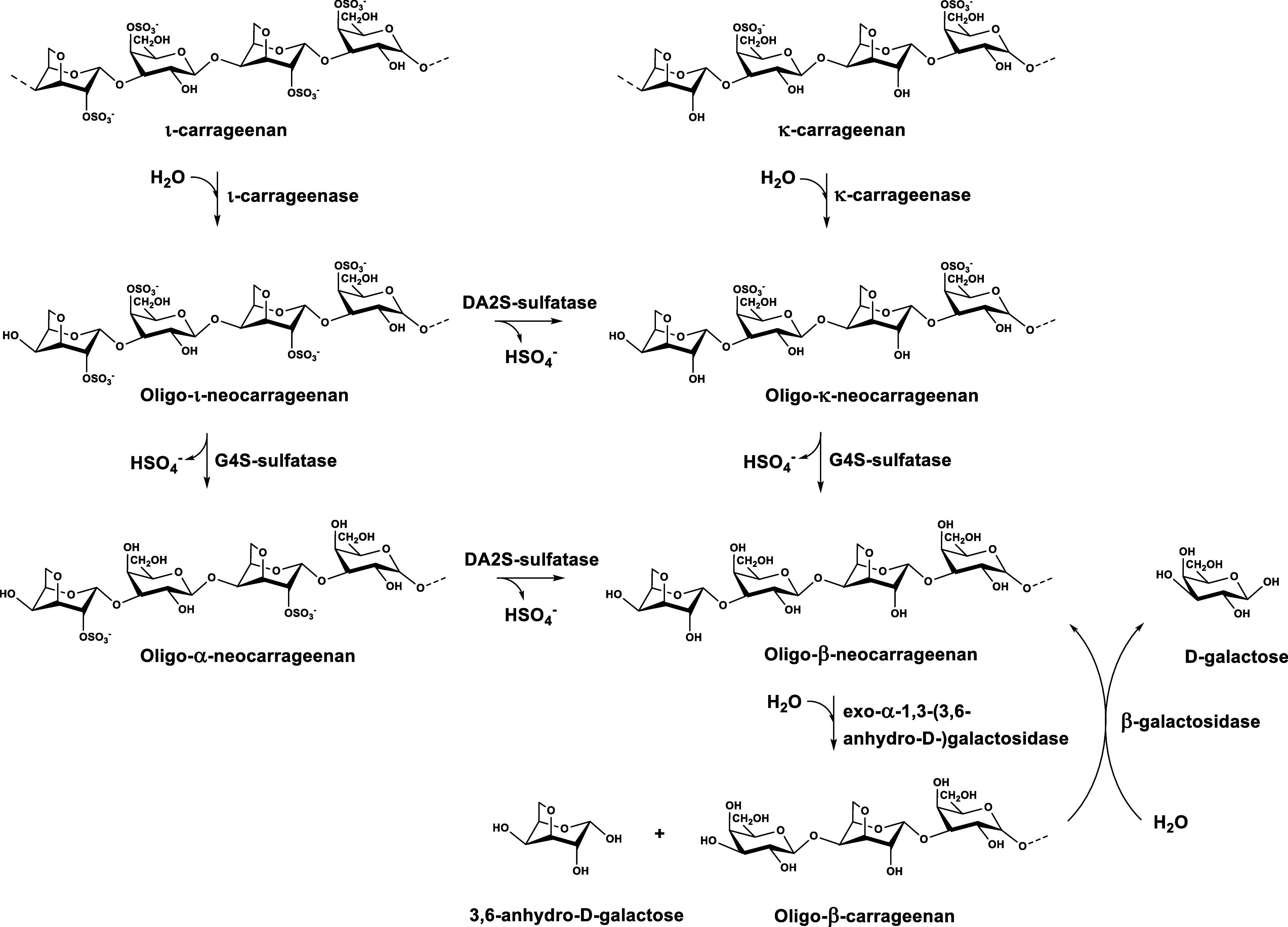
Schematic representation of an in vitro pathway to produce the unique red-algal sugar 3,6-anhydro-D-galactose from ι- and κ-carrabiose containing galactans. Inspired by the carrageenan degradation in marine heterotrophic bacteria, the pathway consists of an initial depolymerization step to yield neocarrageenan oligosaccharides, which are further desulfated by the action of specific sulfatases to form nonsulfated oligosaccharides. These oligosaccharides are further monomerized by a combination of galactosidases to yield DA and D-gal.

Inspired by this natural carrageenolytic pathway in marine bacteria, we tried to design a cell-free in vitro process suitable for the production of tailored carrageenan oligosaccharides and unique red-algal sugar DA by a combination of enzymes from different putative carrageenolytic PULs. This process would therefore consist of an initial depolymerization step yielding neocarrageenan oligosaccharides, a desulfation step to produce nonsulfated intermediates, and a final monomerization to yield DA and D-gal (Figure 2)

For the initial breakdown of carrageenans to even numbered neocarrageenan oligosaccharides, we chose the promiscuous carrageenase from *Bacteroides ovatus* (BovGH16), which accepts both ι - and κ-carrageenan structures and consequently hybrid structures containing those motifs, expanding the accessibility of different galactan substrates.^38,39^ Kinetics of the hydrolysis process revealed that enzymatic degradation of 5 g L^–1^ carrageenan applying 1 U mL^–1^ BovGH16 was completed rapidly within 1 h for κ-carrageenan and 3 h for ι-carrageenan and furcellaran, respectively (Figure S7). In the proceeding steps, the combined action of exo- or endosulfatases and α- and β-galactosidases further hydrolyzes the oligosaccharides to yield DA and D-gal.

### 3.2 Novel Sulfatases for the Utilization of Sulfated Galactans

**Figure 3:**
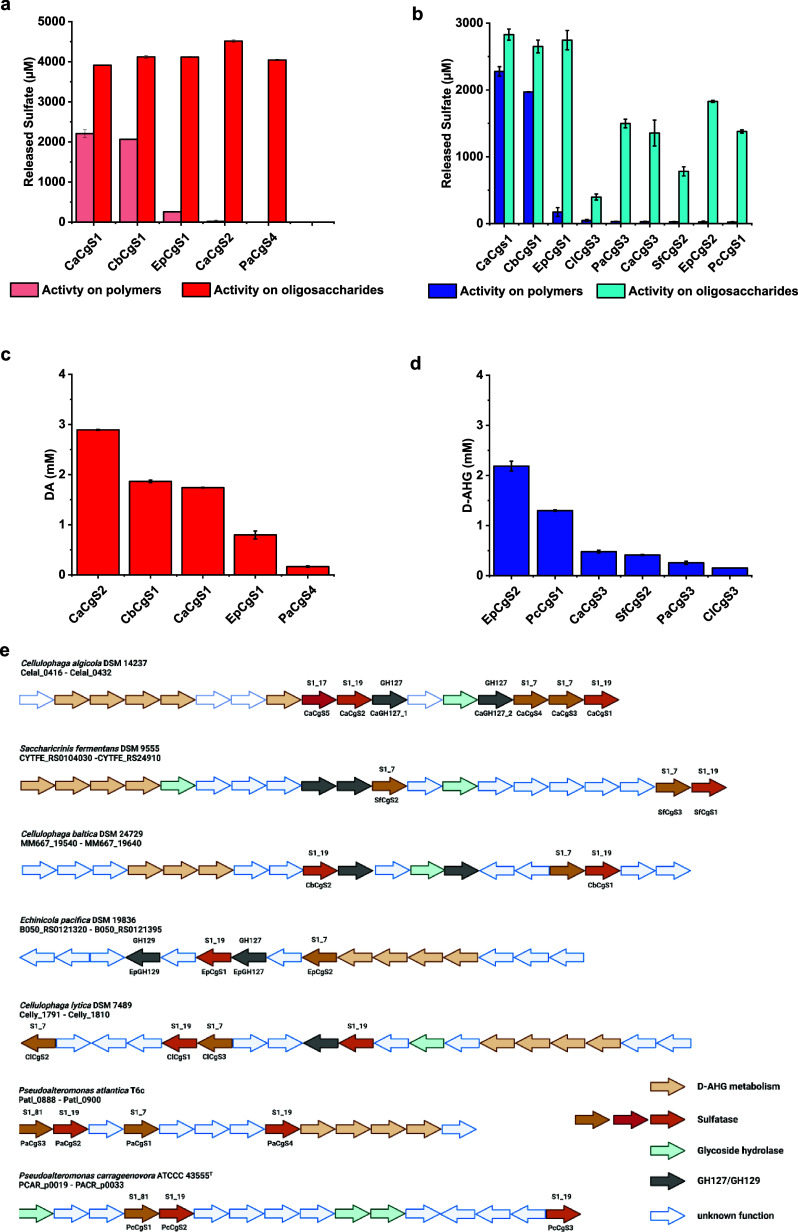
Functional screening of carrageenan sulfatases in carrageenolytic polysaccharide utilization loci (PULs). (a) Sulfatases active on κ-carrageenan or oligo-κ-carrageenan produced by BovGH16 pretreatment. (b) Sulfatases active on ι-carrageenan or oligo-ι-carrageenan produced by BovGH16 pretreatment. The amount of released sulfate was determined by a previously developed sulfate assay.^31^ (c) Production of DA from 0.5% (w/v) κ-carrageenan and (d) production of DA from 0.5% (w/v) ι-carrageenan. For these experiments, the amount of released DA was determined by the action of the DA dehydrogenase from *C. algicola* DSM 14237. (e) Carrageenolytic clusters examined in this study. Descriptions over the arrows indicate the CAZy family (GH127, GH129) or sulfatase classes of the respective enzymes according to the SulfAtlas database.^5^

The hydrolysis of sulfate groups from polymeric carrageenan or its derived oligosaccharides represents the key step in the utilization of sulfated galactans. In order to find a suitable set of sulfatases for this purpose, we aimed to systematically screen several putative carrageenolytic PULs from a wide variety of marine bacteria. This selection was initially based on recently identified PULs from *Pseudoalteromonas carrageenovora* 9^T^ and *Pseudoaltermonas atlantica* T6c.^12,40−42^ Additional PULs were chosen based on the existence of homologues of the carrageenolytic key enzyme exo-(α-1,3)-3,6-anhydro-D-galactosidase from family GH127 or GH129 in these clusters, an approach we already used in a parallel study to identify polysaccharide-active sulfatases.^43^ Analogously, we analyzed the putative carrageenolytic PULs from *Cellulophaga algicola* DSM 14327, *Cellulophaga baltica* DSM 24729, *Cellulophaga lytica* DSM 7489, *Saccharicrinis fermentans* DSM 9555 and *Echinicola pacifica* DSM 19836 to examine the activity of their potential carrageenan sulfatases additionally on oligomeric substrates. The genomic architectures of the investigated clusters are displayed in Figure 3e.

We cloned 22 sulfatase candidates without their N-terminal signal peptides in the pET28a vector for heterologous cytosolic expression and subsequent functional analysis. Since all of the sulfatases belonged to sulfatase family S1 and contained the fGly-signature motif CxPxR, we coexpressed the sulfatase candidates in *E. coli* BL21 (DE3) together with the formylglycine-generating enzyme (FGE) from *Mycobacterium tuberculosis*. From all candidates, we were able to express 16 sulfatases in a soluble form and purify them by affinity chromatography (Figure S1). To characterize the sulfatases, we tested the purified enzymes for sulfatase activity on polymeric ι- and κ-carrageenan and their derived, BovGH16 pretreated oligosaccharides. The application of a previously developed general sulfatase assay, which does not require chromogenic substrates, allowed us to rapidly screen the enzymes for activity toward different carrageenans.^31^

Since κ-carrageenan and κ-neocarrabioses (DA-G4S) are exclusively sulfated at the galactose unit, we directly screened the purified sulfatases toward G4S activity on polymers and crude BovGH16 pretreated oligosaccharides and were able to detect significant activity for five enzymes, all belonging to family S1_19 sulfatases (Figure 3a).^5^ Within these five enzymes, CaCgS1 and CbCgS1 exhibited significant activity on polymeric κ-carrageenan, and we recently found them to be suitable for the enzymatic modification of this sulfated galactan.^43^ EpCgS1, CaCgS2 and PaCgS4, were predominantly active on BovGH16 pretreated κ-carrageenan, while EpCgS1 displayed only little activity toward the polymer. The activity of PaCgS4 toward κ-carrageenan oligosaccharides was demonstrated before.^41^ In order to select a suitable enzyme variant to integrate into the production of DA from carrageenan, we tested the active enzymes for their performance in the proposed in vitro pathway. For this, we used 5 g L^–1^ of κ-carrageenan as substrate and aimed to produce DA by the sequential action of BovGH16, a newly discovered exo-(α-1,3)-3,6-anhydro-D-galactosidase CaGH127_1 and the β-galactosidase WcBGH from *Weizmannia coagulans* DSM 2314, which we both describe below. To enable a rapid screening, we used the 3,6-anhydro-D-galactose dehydrogenase (CaDADH) from *Cellulophaga algicola* DSM 14237, which we found to be exclusively active on DA and allowed us to directly couple the production of this compound to NADH formation. Within this setup, we detected the highest production of DA when we used CaCgS2 for the desulfation step (Figure 3c). Further NMR-studies confirmed that CaCgS2 is an exo-G4S-κ-carrageenan sulfatase (Figure S3).

**Figure 4:**
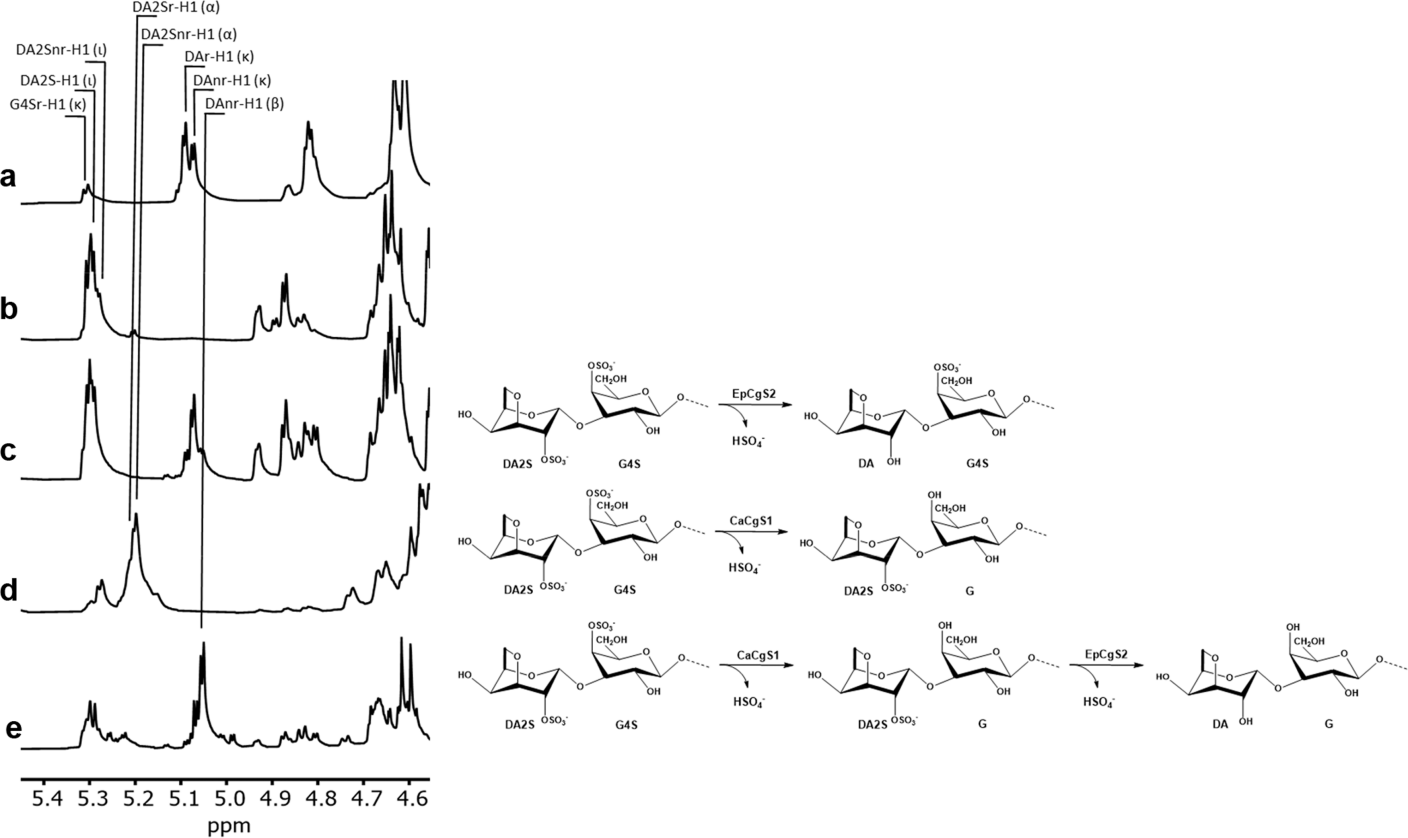
^1^H NMR spectroscopy reveals the mode of action of EpCgS2. (a) κ-carrageenan incubated with BovGH16, yielding oligo-κ-neocarrageenans. (b) ι-carrageenan incubated with BovGH16, yielding oligo-ι-neocarrageenans. (c) ι-carrageenan coincubated with BovGH16 and EpCgS2. Desulfation of oligo-ι-neocarrageenans by EpCgS2 leads to the formation of κ-carrageenan moieties at the nonreducing end, as indicated by the emerging signals for the α-anomeric proton of DAnr-H1 at 5.08 ppm in the ^1^H NMR spectrum. (d) ι-carrageenan coincubated with BovGH16 and CaCgS1. Desulfation of oligo-ι-neocarrageenan by CaCgS1 leads to the formation of α-neocarrageenan moieties, as indicated by the emerging signal for the α-anomeric proton of DA2Snr-H1 at 5.20 ppm in the ^1^H NMR spectrum. (e) ι-carrageenan sequentially incubated with BovGH16, CaCgS1 and EpCgS2. The prior incubation of the BovGH16 generated oligo-ι-neocarrageenans with CaCgS1 leads to the formation of α-neocarrageenan oligosaccharides (5.20 ppm). Subsequent incubation of the reaction product with EpCgS2 further desulfates α-neocarrageenan to β-neocarrageenan oligosaccharides, indicated by the α-anomeric signal DAnr-H1 (β) at around 5.06 ppm. The structures next to the spectra display the respective, sequential sulfatase reactions. The reactions were performed at 37 °C and terminated by heat-inactivation of the enzyme. Enzymes were removed by centrifugation, and the supernatant was dried before resuspension in D_2_O. ^1^H NMR spectra were recorded at 70 °C.

When we analogously tested the sulfatase candidates for the desulfation of ι–carrageenan and BovGH16 pretreated hydrolysates, we observed sulfate release from the polymeric substrate by CaCgs1 and CbCgS1, while seven constructs catalyzed sulfate hydrolysis from oligosaccharides exclusively. However, by this approach, it was not possible to distinguish between sulfate selectivity on the ι-neocarrabiose repeating unit DA2S-G4S, since this motif offers two possible targets for sulfate hydrolysis. In order to selectively screen for DA2S-activity, we additionally integrated these sulfatase candidates in the proposed in vitro pathway, hereby also utilizing the former identified G4S-sulfatase CaCgS2. With this setup, DA production would take place only when a DA2S-sulfatase activity is present that transforms ι-neocarrabioses into κ-neocarrabioses. We were able to identify six sulfatases, distributed across the tested PULs, whose catalytic activity enabled the release of DA from ι-carrageenan. All of the putative DA2S-sulfatases were devoid of activity on polymeric substrates, indicating an exomode of action for these enzymes, and were all classified as S1_7 and S1_81 sulfatases according to the SulftAtlas database.^5^ Thus, CaCgS1 and CbCgs1 are promiscuous carrageenan endo-G4S-sulfatases, which we described in detail in another work,^43^ and EpCgS1 promiscuously hydrolyses G4S moieties in ι- and κ-neocarrageenan oligosaccharides (Figure S4). When we incubated the most active DA2S sulfatase, EpCgS2, with BovGH16 pretreated ι-carrageenan, ^1^H NMR of the crude oligosaccharide products confirmed the formation of κ-neocarrabioses at the nonreducing end, which was indicated by the formation of the characteristic α-anomeric signals of DAnr-H1 (κ) at 5.08 ppm (Figure 4c). To the best of our knowledge, this is the first demonstration of the desulfation of ι-carrabioses to a κ-carrabiose. When we preincubated the ι-neocarrageenan oligosaccharides with the S1_19 endosulfatase CaCgS1 (exhibiting G4S activity on ι-neocarrabiose moieties (DA2S-G4S), thus producing α-neocarrageenan structures (DA2S-G)(Figure 4d)), and subsequently added EpCgS2 to this reaction product, a new α-anomeric proton signal appeared at 5.06 ppm, next to the α-anomeric signals of DAnr-H1 (κ). Simultaneously, the α-anomeric proton signal of DA2S at 5.20 ppm disappeared.^11,44^ These new peaks at around 5.08 ppm are attributed to the formation of β-neocarrabiose moieties (DA-G), essentially resulting from the removal of the sulfate group at the anhydrogalactose of α-neocarrabioses.^12,45^ With this approach, we were able to confirm the same mode of action also for PaCgS3 (S1_81), PcCgS1 (S1_81), CaCgS3 (S1_7), ClCgS3 (S1_7) and SfCgS2 (S1_7) (Figure S5).

Most characterized carrageenan sulfatases were shown to specifically remove sulfate from β-D-galactose in ι- or κ-carrageenan. Within this study, G4S-desulfation was performed exclusively by sulfatases from class S1_19, while all S1_7 sulfatases studied in our work were selective toward the sulfated anhydro-D-galactose. PaCgS3, corresponding *P. atlantica* T6c Patl_0888 sulfatase, was previously shown to exhibit exo-DA2S-activity on oligo-α-carrageenans.^12^ We could demonstrate that its activity was not limited to α-carrageenan oligosaccharides, as desulfation also occurred toward oligo-ι-carrageenans (Figure S5G). As this exo-DA2S-activity is demonstrated universally by sulfatases across the tested carrageenolytic PULs, the direct desulfation of ι- to κ-carrabioses could indicate an additional step occurring in the carrageenan catabolism of diverse marine heterotrophic bacteria, besides the desulfation of α-neocarrabioses to β-neocarrabioses.^11,12^ The sequence similarity of EpCgS2 and the S1_17 sulfatase *zobellia*_3151 from *Z. galactanivorans* Dsij^T^, which was the first carrageenan sulfatase demonstrating desulfation of oligo-α-carrageenans, is only 37.3%, and interestingly, despite EpCgs2 (S1_7) and PcCgS1 (S1_81) catalyzing the same reaction, they share only 27% identity on amino acid level. Another S1_81 family member, S1_NC from *Pseudoalteromonas fuliginea* PS 47, was believed to catalyze the here-described 2S-activity as it was indicated by structural analysis. However, this activity could not be proven experimentally, presumably due to failed or incomplete fGly-maturation.^38^

**Figure 5:**
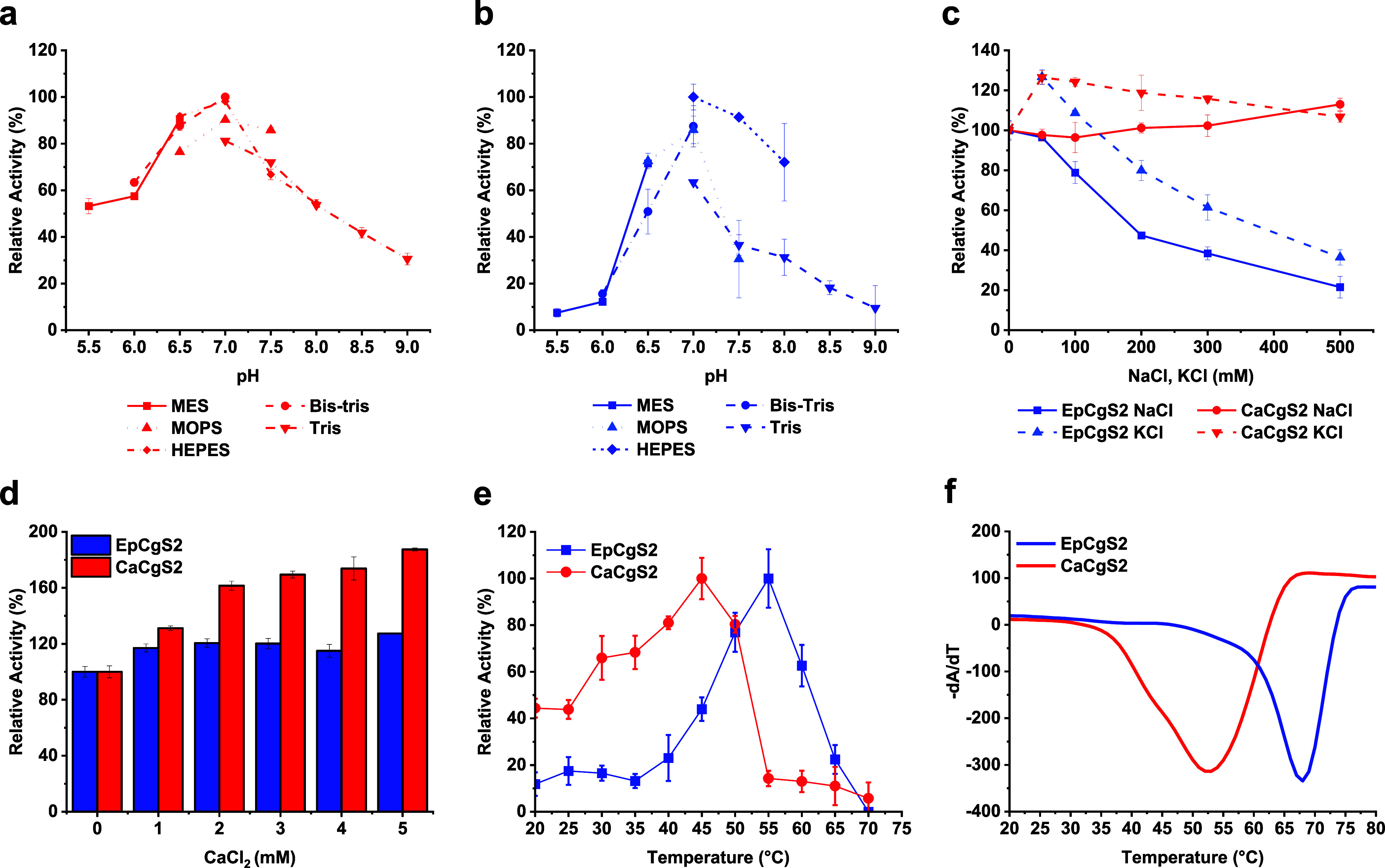
Biochemical Characterization of CaCgS2 and EpCgS2. pH-profiles of CaCgS2 (a) and EpCgS2 (b) at pH values ranging from 5.5 to 9.0 in 20 mM different buffer systems at 37 °C. (c) Tolerance of CaCgS2 and EpCgS2 toward NaCl and KCl in 20 mM Tris-HCl at 37 °C. (d) Effect of CaCl_2_ on the enzymatic activity of EpCgS2 and CaCgS2 at 37 °C in 20 mM Tris-HCl pH 7.5. (e) Temperature profile of CaCgS2 and EpCgS2 ranging from 20 to 70 °C in 20 mM Tris-HCl pH 7.5. (f) The thermodynamic stability of CaCgS2 and EpCgS2 in 20 mM Tris-HCl pH 7.5, determined by Thermofluor and expressed as the melting temperature *T*_m_.

As EpCgS2 and CaCgS2 have demonstrated superior suitability for DA production, we conducted a further biochemical characterization of these enzymes (Figure 5). We determined the pH and buffer optima for both enzymes at pH 7.0 in Bis-Tris buffer. CaCgS2 exhibited notable salt tolerance in both NaCl and KCl within the tested range of 0 to 500 mM. In contrast, EpCgS2 demonstrated activation up to 50 mM KCl but gradually lost activity at higher salt concentrations, showing a similar behavior in the presence of NaCl. The addition of calcium up to 5 mM resulted in an almost two-fold increase in CaCgS2 activity, while the activation of EpCgS2 was comparatively less pronounced. We determined a melting point of 53 °C and an optimal reaction temperature of 45 °C for CaCgS2, with a sharp decline observed as the temperature increased to 55 °C. EpCgS2 exhibited a high melting temperature of 68 °C, and reached maximum activity at 55 °C, which dropped significantly to only 20% at 40 and 65 °C.

### 3.3 Selection of Further Enzymes for Carrageenan Bioconversion

For the further utilization of nonsulfated oligosaccharides, previous studies revealed that the action of exo-(α-1,3)-3,6-anhydro-D-galactosidases from family GH127 and GH129 release DA at the nonreducing end.^11^ Therefore, we cloned, expressed, and tested several GH127/GH129 enzymes encoded in the examined PULs to find a suitable candidate for DA release. We selected the previously characterized ZgGH129 from *Z. galactanivorans* Dsij^T^, and additionally expressed four so far uncharacterized exo-(α-1,3)-3,6-anhydro-D-galactosidases from *C. algicola* DSM 14237 (CaGH127_1 and CaGH127_2), and from *E. pacifica* DSM 19836 (EpGH127 and EpGH129).^11^ We confirmed their activity as all of the tested enzymes were able to release DA from neocarrageenan oligosaccharides after incubation with the respective sulfatases, and all were devoid of activity on sulfated substrates. However, the characterization of these enzymes with their natural substrate is difficult due to β-neocarrabiose (β-NC2) not being commercially available. Recently, Wallace et al. developed a synthetic substrate for the characterization of exo-(α-1,3)-3,6-anhydro-D-galactosidase mimicking the natural α-1,3-linkage, that was modified with a chromogenic functional group.^32^ We synthesized this molecule but detected activity on this substrate only by the two GH129 enzymes and not by GH127 hydrolases, which could indicate a different reaction mechanism of these hydrolases. Alternatively, we directly determined the suitability and performance of the single exo-(α-1,3)-3,6-anhydro-D-galactosidases in the pathway setup by starting the cascade from polymeric κ-carrageenan and coupling DA release to NADH formation by the action of CaDADH. Here, we found rapid NADH formation for CaGH127_1, indicating rapid DA release, while the other candidates showed only low activity within the tested time window (Figure S6A).

We further aimed to increase the yield of DA by hydrolysis of D-gal from the nonreducing end of the oligosaccharide chains after CaGH127_1 action, as it was previously shown that β-galactosidases interplay with exo-(α-1,3)-3,6-anhydro-D-galactosidase during carrageenan utilization.^11^ For this, we tested and characterized the β-galactosidase from *Weizmannia coagulans* DSM 2314 to release D-galactose from the nonreducing end of oligosaccharides, which would make another DA molecule accessible to exo-(α-1,3)-3,6-anhydro-D-galactosidase action. Within this setup, we confirmed increased DA production (Figure S6B).

### 3.4 Demonstration of the Bioconversion of Sulfated Galactans

By harnessing a combination of a promiscuous carrageenase, two novel carrageenan sulfatases, and two hydrolases for the release of DA and D-gal, we were able to set up a complete enzymatic process for the utilization of different types of carrageenans. We tried to enhance the cascade performance with respect to different process parameters and enzyme ratios. Optimization of reaction pH and temperature revealed an optimal pH for DA production between 6.5 and 7.0 at 35 °C. The optimal enzyme amounts were determined to be 0.01 mg mL^–1^ WcBGH, 0.5 mg mL^–1^ CaCgS2, 0.03 mg mL^–1^ Ca127_1, and 0.4 mg mL^–1^ EpCgS2 (when hydrolyzing ι-carrageenan) (Figure S11).

Finally, we tested the applicability of the enzyme cocktail on different DA-containing carrageenans and natural hybrid structures in order to verify the versatility of the system toward structurally different sources. For this, we selected a variety of sulfated galactans, including the κ/β-hybrid carrageenan furcellaran, as well as a ι/κ-hybrid carrageenan extracted from gametophytes of the red algae *Chondrus crispus*. Aqueous extraction of ι- and κ-carrageenan from the red algae *Eucheuma spinosum* and *Κappaphycus alvarezii* yielded hybrid ι/ν- and κ/μ-hybrid-carrageenans, respectively (Figure S12).

In a proof of concept, we tested the optimized enzyme mix for DA production toward the carrageenans at a nongelling concentration of 5 g L^–1^ to avoid diffusion limitations.

**Figure 6:**
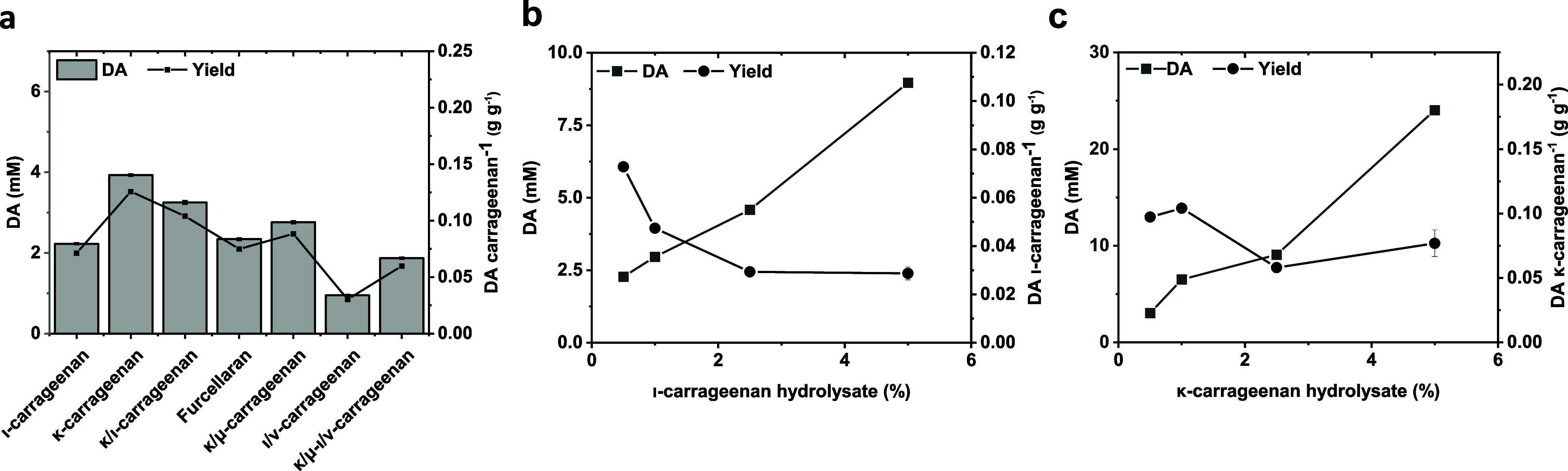
Bioconversion of different sulfated galactans for the production of DA. (a) Production of DA from 0.5% (w/v) of the indicated carrageenans determined after 16 h reaction at 37 °C. (b) Production of DA applying different concentrations of BovGH16 prehydrolyzed ι-carrageenan after 16 h at 37 °C. (c) Production of DA applying different concentrations of BovGH16 prehydrolyzed κ-carrageenan after 16 h at 37 °C.

After 16 h, we confirmed the production of DA from all tested sources (Figure 6a). The highest yield of DA was produced from κ-carrageenan at a final concentration of 3.9 mM, referring to 0.126 g DA per gram of substrate. Assuming a theoretical DA content in κ-carrageenan between 28 and 35%, the yield of DA would be between 35 and 48%. The second highest titer was observed when we applied the κ/ι-hybrid carrageenan extracted from *C. crispus* with 3.25 mM DA. Converting furcellaran and the κ/μ-hybrid-carrageenan extracted from *K. alvarezii*, we produced 2.35 and 2.76 mM of DA, corresponding to 0.075 and 0.09 g g^–1^, respectively. The amount of DA released from ι-carrageenan was in a similar range with 2.23 mM (0.072 g g^–1^), while we obtained the lowest yield from hybrid ι/ν-carrageenan (0.95 mM, corresponding to 0.031 g g^–1^). We could achieve a slightly higher yield of DA from aqueous extracted κ/ι-carrageenan, additionally containing μ- and ν-carrabioses, with 1.87 mM (0.06 g g^–1^).

Only recently, the first enzymatic production of DA was demonstrated from κ-carrageenan by application of the cell-free extract of *C. echini* A3^T^ to the prehydrolyzed galactan in a three-step process with intermediate enzyme inactivation.^27^ In this approach, a final titer of 0.24 g of DA was achieved from 1 g of κ-carrageenan starting from 5 g L^–1^ polymer. In our one-pot and cell-free approach, we produced up to 0.13 g of DA per 1 g of κ-carrageenan, which emphasizes the complex process of carrageenan degradation and indicates the necessity of additional hydrolytic enzymes (as they are likely present in the cell lysate of *C. echini* A3^T^) for the complete bioconversion of carrageenans. This putative action of other hydrolytic enzymes is emphasized by the low yield of DA from ι – carrageenan. With 0.072 g g^–1^, only 25 to 30% of theoretically available DA was released in this case. When we increased the substrate load of κ- and ι-carrageenan by applying prehydrolyzed galactans to overcome gelation of the reaction media, we could achieve up to 24 mM (3.9 g L^–1^) and 9 mM (1.5 g L^–1^) of DA from 5% (w/v) hydrolysates after 16 h at 37 °C, respectively (Figure 6b,c). However, the increase of titer was not linear to the increase of substrate load, and for the highest load tested, the yield decreased to 0.08 and 0.03 g g^–1^ substrate for κ- and ι-carrageenan, respectively, indicating potential stability or inhibition problems of the enzymes.

The reduced yield of DA from the hybrid carrageenans is an expected consequence of the presence of noncyclized μ- and ν-precursor moieties that would represent an enzyme-resistant fraction to BovGH16. To the best of our knowledge, up to now, no carrageenase is able to hydrolyze ν- or μ-carrageenans, and there are no reports on sulfatases capable of removing sulfate from these structures to yield D-gal. The low yield of DA from ι -carrageenan can further be a consequence of several additional factors, including the incomplete hydrolysis of the polymer by the initial carrageenase and probably the action of more sulfatases supporting the utilization of ι -carrageenan in marine bacteria. To overcome this issue, an extensive analysis of the cascade intermediates and, in general, a more detailed analysis of the complete carrageenan utilization in marine bacteria will be necessary in future investigations.

In conclusion, we successfully set up a comprehensive enzymatic process for the conversion of sulfated galactans to DA by the systematic functional evaluation of known and putative carrageenolytic PULs, including an in-depth characterization of promising enzymes. Particularly the identification and characterization of novel carrageenan sulfatases, including a so far undescribed exo-DA2S-activity toward ι-carrageenan, not only enhance our understanding of carrageenan utilization in marine bacteria but also lay the groundwork for sustainable production processes of valuable red-algal sugars and customized oligosaccharides. The combination of an initial carrageenase-driven depolymerization step with further desulfation by two novel exoacting sulfatases and a final monomerization of carrageenan-derived oligosaccharides applying two galactosidases was successfully demonstrated for a broad variety of sulfated galactans, highlighting the versatility of the developed process. However, the efficiency of the process was found to strongly depend on the structure of the used carrageenan substrates, resulting in low DA yields, especially when hybrid carrageenans were applied. Hence, a comprehensive analysis of intermediate reaction products and subsequent process modifications is imperative for future investigations and will contribute to the sustainable production of valuable, carrageenan-derived products.

## Supporting Information Available

List of genes and primers used in this study, SDS-PAGE of proteins purified in this study, phylogenetic tree of the S1_19, S1_7, and S1_81 sulfatases used in this study, ^1^H NMR of CaCgS2 reaction products, ^1^H NMR of EpCgS1 reaction products, ^1^H NMR of the reaction products of exo-DA2S-sulfatases identified in this study, selection of further hydrolases for carrageenan degradation, hydrolysis of carrageenans by BovGH16, biochemical characterization of CaDADH, biochemical characterization of WcBGH, optimization of cascade performance, and structures and ^1^H NMR spectra of extracted carrageenans used in this study (PDF)

## Abbreviations

D-gal: D-galactose
DA: 3,6-anhydro-D-galactose
CgS: carrageenan sulfatase
fGly: formylglycine
G4S: galactose-4-sulfate
DA2S: 3,6-anhydro-D-galactose-2-sulfate
FGE: formylgycine-generating enzyme
GH: glycoside hydrolase
DH: dehydrogenase
pNP: para-nitrophenol
BGH: β-galactosidase
DNS: 3,5-dinitrosalicylic acid
