# Supplementary material for: Biocatalytic Conversion of Carrageenans for the Production of 3,6-Anhydro-D-galactose
Source: J Agric Food Chem. 2024 Mar 5;72(11):5816–27. doi: 10.1021/acs.jafc.3c08613 (PMC10958521; doi:10.1021/acs.jafc.3c08613)
Supplement: Supplementary file 1 — jf3c08613_si_001.pdf [file jf3c08613_si_001.pdf]

Supporting Information

Biocatalytic conversion of carrageenans for the production of 3,6-anhydro-D-galactose

Alexander Fuchs[1], Dennis Romeis[1], Enrico Hupfeld[1] & Volker Sieber [1,2,3,4,*]

[1] Chair of Chemistry of Biogenic Resources, Technical University of Munich, TUM Campus Straubing for Biotechnology and Sustainability, Schulgasse 16, 94315, Straubing, Germany

[2] SynBioFoundry@TUM, Technical University of Munich, Schulgasse 22, 94315 Straubing, Germany

[3] Catalytic Research Center, Ernst-Otto-Fischer-Straße1, 85748, Garching, Germany

[4] School of Chemistry and Molecular Biosciences, The University of Queensland, 68 Copper Road, St. Lucia 4072, Australia

Volker Sieber*: sieber@tum.de

Tel.: +49 (0) 9421 187-300

Fax: +49 (0) 9421 187-310

Table S1: List of primers and restriction enzymes to clone the enzymes used in this study.

Gene Locus	Protein_ID	Protein Name	Organism	Forward Primer	Reverse Primer	Restriction sites	Expression
**Sulfatases**							
Celal_0432	WP_013549269.1	CaCgS1	*Cellulophaga algicola* DSM 14237	TATATAGCTAGCATGATTAATGCTCAAAGTCAGACAG	TATATACTCGAGTTACTTTTGCTCAAGTTTAAAAGTATC	NheI/XhoI	+
Celal_0430	WP_013549267.1	CaCgS2	*Cellulophaga algicola* DSM 14237	TATATAGCTAGCAATCAACCGACAAAGAAAAAACC	TATATACTCGAGCTATTTTTTTAGAATTTTTTTTACAA	NheI/XhoI	+
Celal_0431	WP_013549268.1	CaCgS3	*Cellulophaga algicola* DSM 14237	TATATAGCTAGCAAAGAAAATGAAGTCAAAAAAGAGG	TATATACTCGAGCTAGTTACTTGAAGTTGATAAATTAC	NheI/XhoI	+
Celal_0424	WP_013549261.1	CaCgS4	*Cellulophaga algicola* DSM 14237	TAT ATA GGT CTC ACA TGC AAA AAA GAC CGA ATG	TAT ATA GGT CTC TCT TAG GGT AAA ACA TCA ATT C	BsaI	-
Celal_0425	WP_013549262.1	CaCgS5	*Cellulophaga algicola* DSM 14237	TAT ATA CAT ATG CAA GAA AAA CCA AAC ATT ATT TT	TAT ATA CTC GAG TTA ATA TTT TAA AAG ATT TGG TC	NdeI/XhoI	+
Celly_1796	WP_013621364.1	ClCgS3	*Cellulophaga lytica* DSM 7489	TATATACATATGAACAACAAACCTAAAAAAGTTGC	TATATACTCGAGTTATTTAGGTAATGCAAAGGTAT	NdeI/XhoI	+
Celly_1791	WP_013621359.1	ClCgS2	*Cellulophaga lytica* DSM 7489	TATATACATATGTATTCTCAAAAAAAACCTAATATAC	TATATACTCGAGTTACTTTAATTTTGGTTTATTTCC	NdeI/XhoI	-
Celly_1795	WP_013621363.1	ClCgS1	*Cellulophaga lytica* DSM 7489	TATATACATATGCAATCACAAAAAGAGACAAAAAAAG	TATATACTCGAGTTAGTTAAGTTGTGCTAAATTGCC	NdeI/XhoI	-
CYTFE_RS24910	WP_044212231.1	SfCgS1	*Saccharicrinis fermentans* DSM 9555	TATATACATATGACGAAAATAAGAAAGTCTCAACC	TATATACTCGAGTTATTTTCTACTCTCTAACACTTTTTC	NdeI/XhoI	-
CYTFE_RS0104085	WP_044212247.1	SfCgS2	*Saccharicrinis fermentans* DSM 9555	TATATACATATGGTTCAAGTAAAGCAAGAAGATAAG	TATATACTCGAGTCAATGGGACATTGCAGACTTCTC	NdeI/XhoI	+
CYTFE_RS0104125	WP_027470782.1	SfCgS3	*Saccharicrinis fermentans* DSM 9555	TATATAGCTAGCAATGTTAAATCAGTACAACATGAG	TATATACTCGAGCTACTTTATCTCAGCCATATTACCC	NheI/XhoI	+
B050_RS0121345	WP_018475998.1	EpCgS1	*Echinicola pacifica* DSM 19836	TATATACATATGGACCAGCGGCCAAACATTCTCG	TATATACTCGAGTTACTTAATCTTGAATGTGTCATTAAAAG	NdeI/XhoI	+
B050_RS0121360	WP_018476001.1	EpCgS2	*Echinicola pacifica* DSM 19836	TATATACATATGGGTACTCAGCCCAATATCCTCTTTATC	TATATACTCGAGTTAGAATAAATCCTGACTAAGTTGC	NdeI/XhoI	+

M666_RS19540	WP_029445533.1	CbCgS1	*Cellulophaga baltica* DSM 24729	TAT ATA CAT ATG TGT AAA GAA AAA GAA AAA AAT ACC ACT G	TAT ATA CTC GAG TTA GTC TTG TGA TAT TTT AAA AGT TTG	NdeI/XhoI	+
M666_RS19500	WP_039326103.1	CbCgS2	*Cellulophaga baltica* DSM 24729	Ordered codon-optimized from GENEART AG, Regensburg, Germany		BsaI	+
Patl_0891 [1]	WP_011573777.1	PaCgS1	*Pseudoalteromonas atlantica* T6c	TAT ATA GGA TCC GTA CCA AAG AAC ACT GC	TAT ATA CTC GAG TCA TCG TGT TTG TCC TGC G	BamHI/XhoI	-
Patl_0889 [2]	WP_011573775.1	PaCgS2	*Pseudoalteromonas atlantica* T6c	TAT ATA GCT AGC GAT GCT GGG CAA AGT AAA G	TAT ATA CTC GAG TTA TTT GTT GTT TTC AAA ATA AAG TGG	NheI/XhoI	+
Patl_0888 [3]	WP_011573774.1	PaCgS3	*Pseudoalteromonas atlantica* T6c	TAT ATA GCT AGC AAT GAA CCA GAG CAA GAA AC	TAT ATA CTC GAG TTA GTC AAC GTT ATA GGC TTC	NheI/XhoI	+
Patl_0895 [2]	WP_011573781.1	PaCgS4	*Pseudoalteromonas atlantica* T6c	TAT ATA CAT ATG CAA CCG AAT ATC GTT TTT CTG	TAT ATA CTC GAG TTA TTC TAA GCG TTT TGG TAC G	NdeI/XhoI	+
PCAR9_P0022	WP_104644202.1	PcCgs1	*Pseudoalteromonas carrageenovora* ATCC 43555^T	TAT ATA GGA TCC GAT GCA GGG CTG AAA AAC T	TAT ATA CTC GAG CTA TAA AAA ATC TTT CAT GTG GTG	BamHI/XhoI	+
PCAR9_P0023	WP_104644203.1	PcCgs2	*Pseudoalteromonas carrageenovora* ATCC 43555^T	TAT ATA CAT ATG GAA CAA AAA CCC AAT ATT ATT CTT ATT G	TAT ATA CTC GAG TTA CTT AGT TGT TTT AAA CGT TTT ATC	NdeI/XhoI	+
PCAR9_P0034	WP_104644210.1	PcCgs3	*Pseudoalteromonas carrageenovora* ATCC 43555^T	TAT ATA CAT ATG ACA GAA AAA CCT AAT ATT GTT TTA ATT TTT GC	TAT ATA CTC GAG TTA AGG CTG AGT GGC TGG	NdeI/XhoI	-
**exo-(α-1,3)-3,6-anhydro-D-galactosidases**							
B050_RS0121350	WP_018475999.1	EpGH127	*Echinicola pacifica* DSM 19836	TAT ATA GCT AGC ATG AGC AAT AAA ATT GGA AAT ACA G	TAT ATA CTC GAG TTA ATT TAT GTT CCA CAC TAC C	NheI/XhoI	+
B050_RS0121335	WP_018475996.1	EpGH129	*Echinicola pacifica* DSM 19836	AAA AAA GGT CTC ACA TGG AGT CCA CGG GAG AAA TCA AAA A	TTT TTT GGT CTC TCT TAC TAT TCC ATG GTT AGT GTC ATT TGG	BsaI	+
Celal_0426	WP_013549263.1	CaGH127_1	*Cellulophaga algicola* DSM 14237	AAA AAA GGT CTC ACA TGA AAC AAG AGG TAA AAG AT	TTT TTT GGT CTC TCT TAT TTT TGC TCC CAT ACA AC	BsaI	+
Celal_0429	WP_013549266.1	CaGH127_2	*Cellulophaga algicola* DSM 14237	AAA AAA GGT CTC ACA TGA ATA CCA CCA TGA AAA AA	TTT TTT GGT CTC TCT TAT TCC CAG ACT ACG GGC AT	BsaI	+
Zgal_3152 [4]	WP_013994483.1	ZgGH129	*Zobellia galactanivorans* Dsij^T	AAA AAA GGT CTC ACA TGT TTG ACT CCA TTA GCC	TTT TTT GGT CTC TCT TAT TAA TCC ATA TGT AAA CTC ACT TG	BsaI	+
**β-galactosidases**							

CYJ15_RS03035	WP_110133817.1	WcBGH*	*Weizmannia coagulans* DSM 2314	CAATTGAATTCGATGTTAAAAAACAAGAAAAATTTTATTATG	GATATGTCGACCTATTTTTCAATTACCTGCAAAATTTTCA	EcoRI/SalI	+
**3,6-anhydro-d-galactosedehydrogenase**							
Celal_0418	WP_013549255.1	CaDADH	*Cellulophaga algicola* DSM 14237	AAA AAA GGT CTC ACA TGA AAA CAA ATT TAA AAT TC		BsaI	+
**Carrageenases**							
EIY64570 [5]		BovGH16	*Bacteroides ovatus* CL02T12C04	Ordered codon-optimized from GENEART AG, Regensburg, Germany		BsaI	+

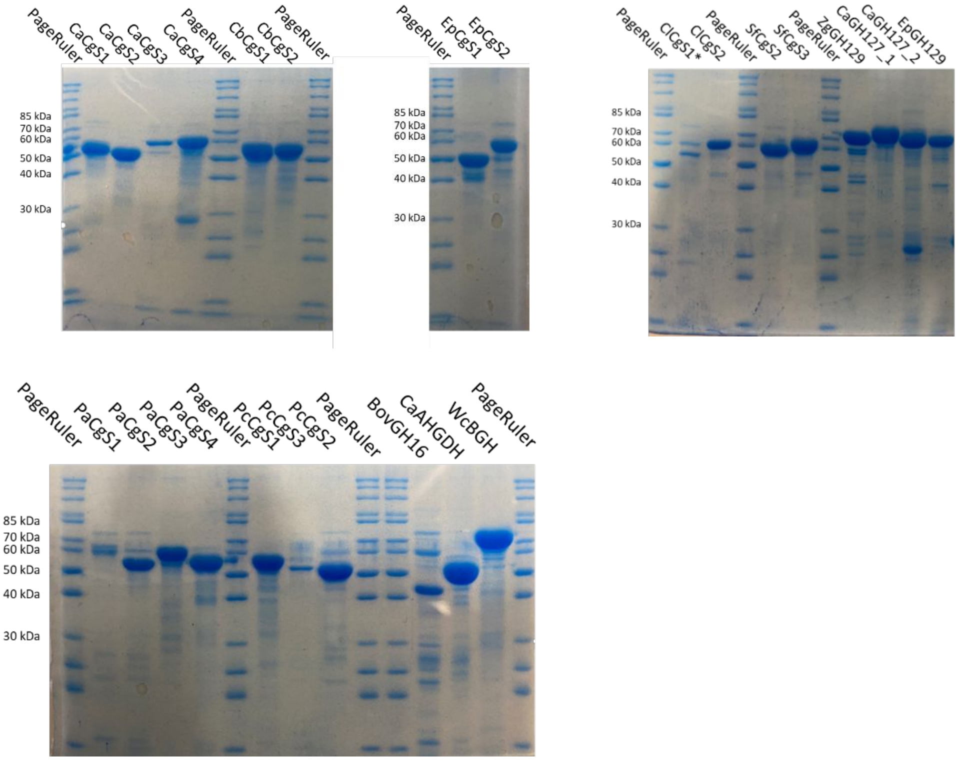

Figure S1: Soluble proteins produced from the carrageenolytic PULs after purification by nickel-affinity chromatography. PageRuler: Thermo Scientific PageRuler Unstained Protein Ladder. ClCgS1*: Protein started to precipitate after IMAC purification.

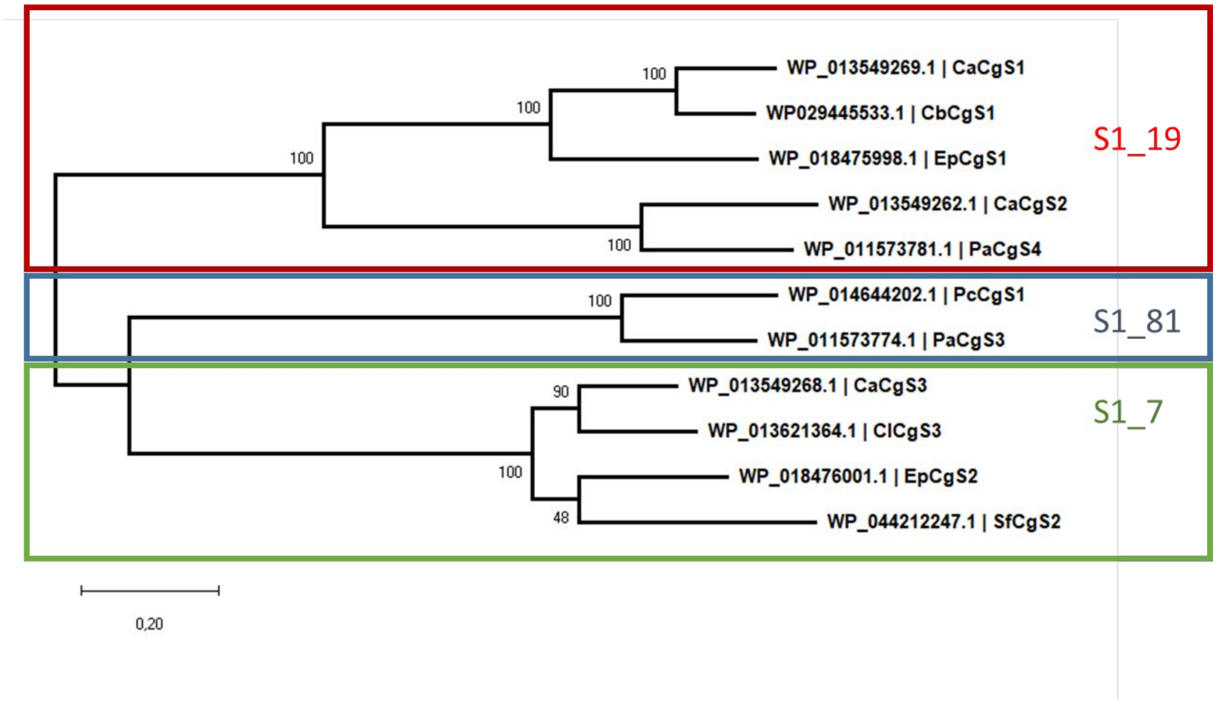

Figure S2: Phylogenetic tree of the characterized carrageenan sulfatases from the studied carrageenolytic PULs and their respective sulfatase families: Red: S1_19 sulfatases, Blue: S1_81 sulfatases, Green: S1_7 sulfatases. The sequence alignment and phylogenetic tree construction were performed using MEGA-X software[6]. Sequence alignment was performed using the MUSCLE algorithm and the phylogenetic tree was developed using Maximum Likelihood algorithm embedded in MEGA-X using default parameters [7, 8]. The sulfatase families were annotated using the SulfAtlas database [9].

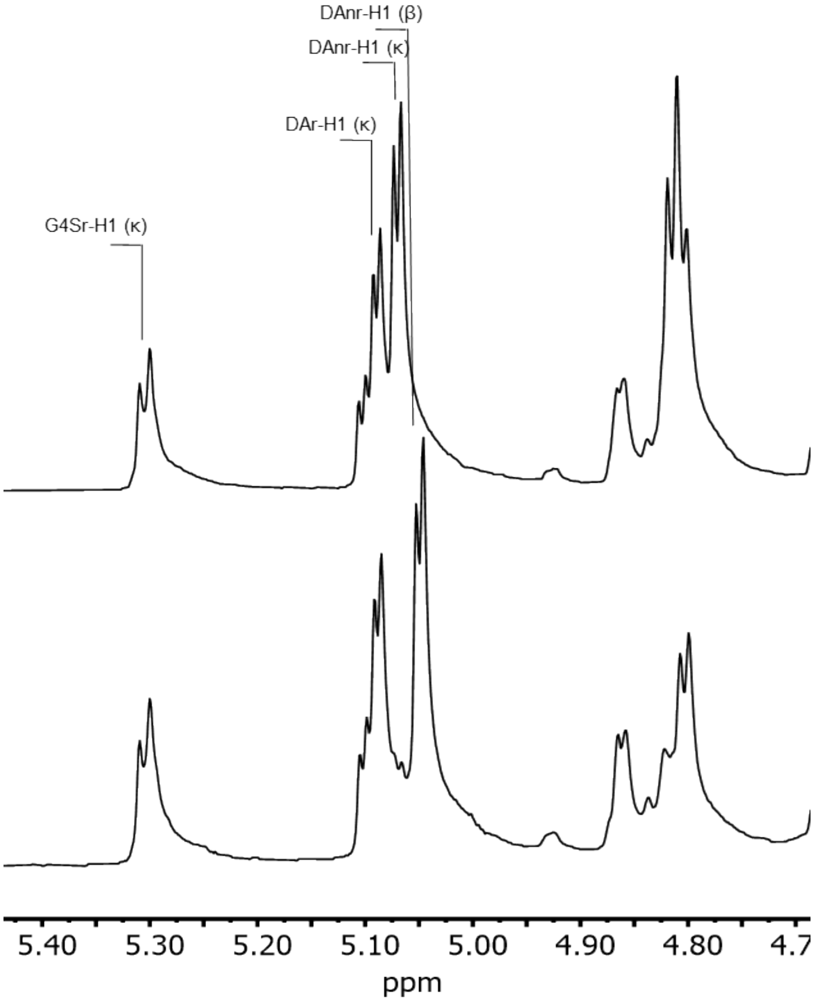

Figure S3: ^{1}H-NMR reveals the mode of action of CaCgS2. Spectrum of the reaction product of CaCgS2 (bottom) after incubation with κ-neocarratetraose (top). After incubation of the oligosaccharide with CaCgS2, the α-anomeric signals of DAnr-H1 are shifted from 5.08 to 5.06 ppm, indication the generation of β-carrageenan moieties on the non-reducing end.[10] Spectra were recorded at 70 °C.

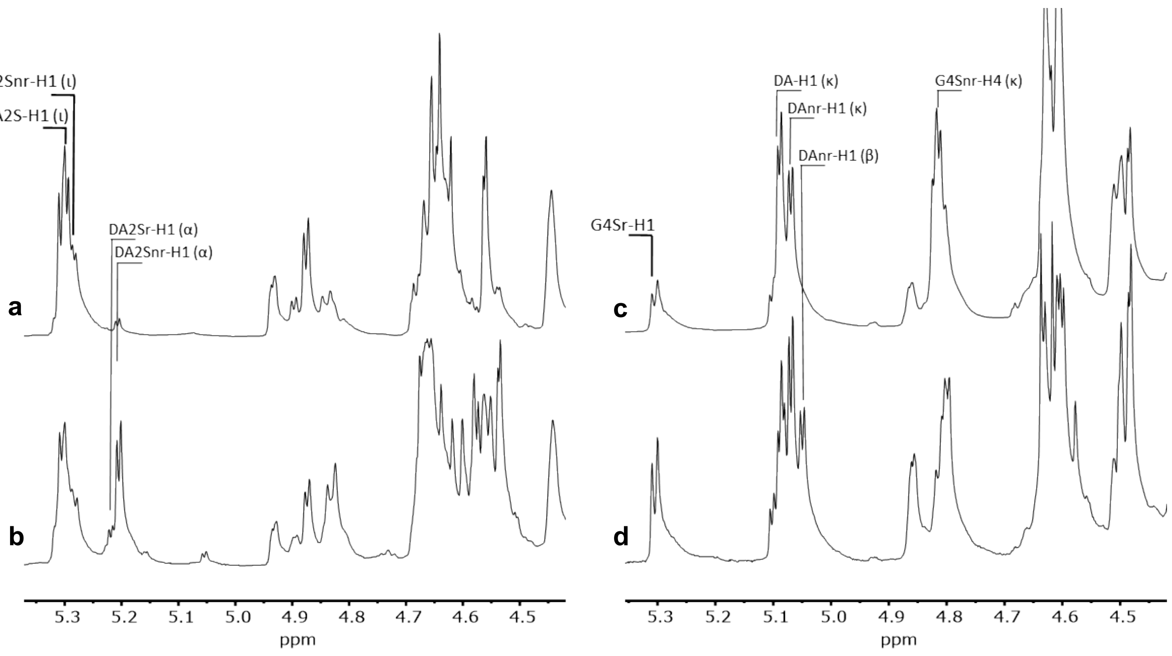

Figure S4: ^{1}H-NMR confirms EpCgS1 to be a promiscuous sulfatase acting on ι- and κ-carrageenan oligosaccharides. After incubation of ι-carrageenan with BovGH16 (**a**) and EpCgS1 (**b**), new α-anomeric signals appear at around 5.20 ppm, indicating the production of α-carrageenan motifs (DA2Snr). When BovGH16 pre-treated κ-carrageenan oligosaccharides (**c**) are incubated with EpCgS1 (**d**), the sulfatase promotes the formation of β-carrabioses and the production of oligo-κ/β-carrageenan, which is confirmed by new α-anomeric proton signals at around 5.06 ppm attributed to DAnr-H1. Spectra were recorded at 70 °C.

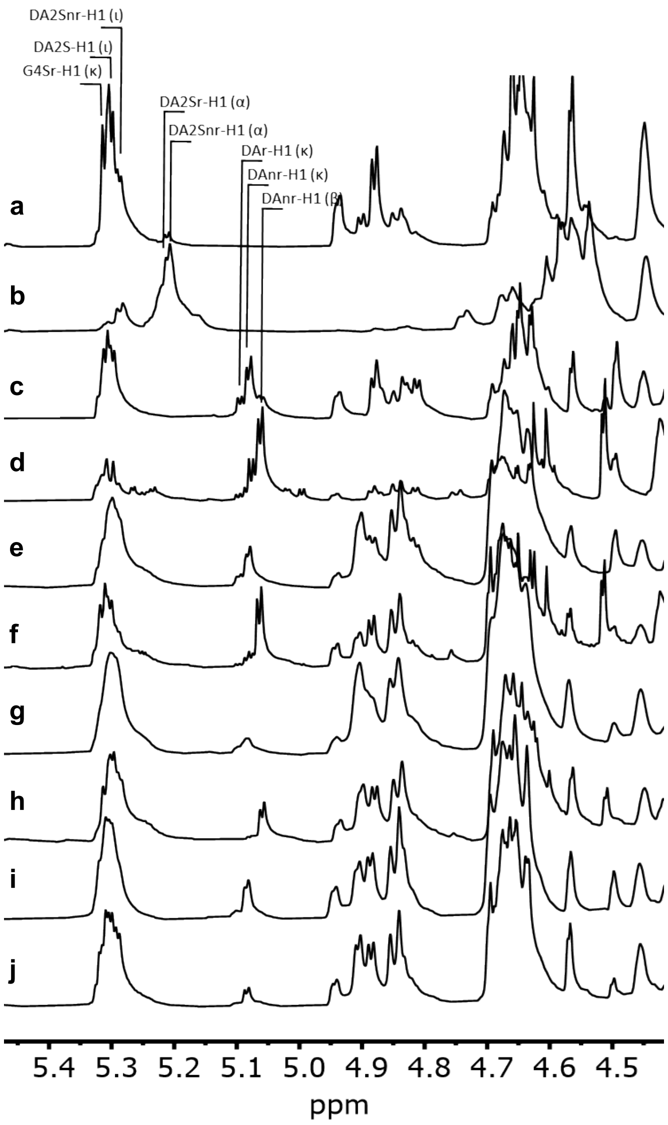

Figure S5: ^{1}H-NMR of the reaction products of the exo-DA2S-sulfatases from sulfatase family S1_7 and S1_81 on crude ι-carrageenan oligosaccharides. After incubation of ι-carrageenan with BovGH16 and the respective S1_7/S1_81 sulfatases, new α-anomeric signals appear at around 5.08 ppm, revealing the desulfation on position 2 of DA and the production of κ-carrageenan motifs. When the oligosaccharides are pre-incubated with CaCgS1, producing α-carrabiose moieties (5.20 ppm), subsequent incubation with the S1_7/S1_81 sulfatases promotes the formation of β-carrabioses, which is confirmed by new α-anomeric signals at 5.06 ppm. (**a**) ι-carrageenan incubated with BovGH16. (**b**) ι-carrageenan incubated with BovGH16 and CaCgS1. (**c**) ι-carrageenan incubated with BovGH16 and CaCgS3. (**d**) ι-carrageenan incubated with BovGH16, CaCgS1, and CaCgS3. (**e**) ι-carrageenan incubated with BovGH16 and PcCgS1. (**f**) ι-carrageenan incubated with BovGH16, CaCgS1, and

PcCgS1. (**g**) ι-carrageenan incubated with BovGH16 and PaCgS3. (**h**) ι-carrageenan incubated with BovGH16, CaCgS1, and PaCgS3. (**i**) ι-carrageenan incubated with BovGH16 and SfCgS2. (**j**) ι-carrageenan incubated with BovGH16 and ClCgS3. Spectra were recorded at 70 °C.

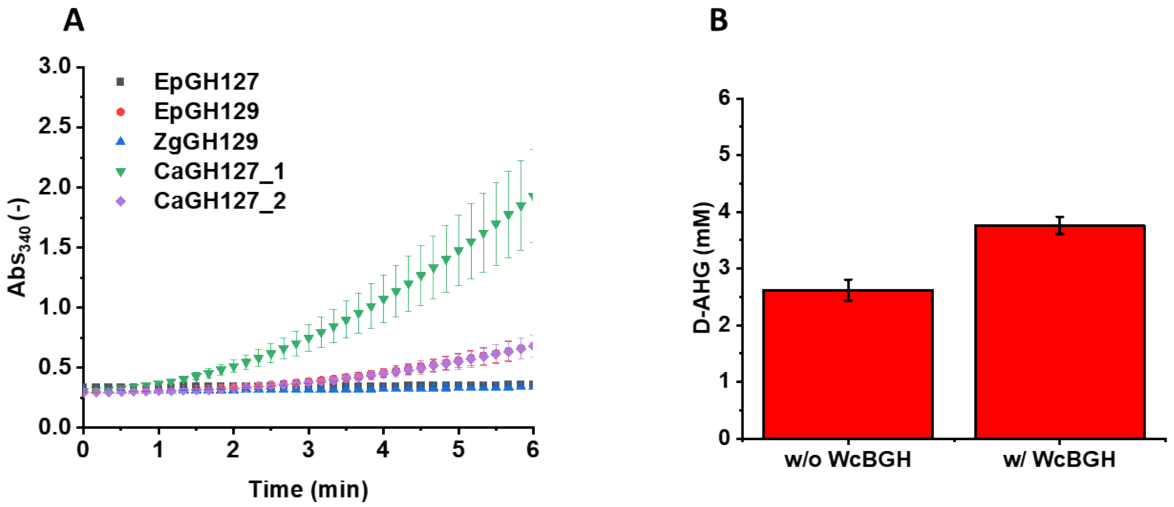

Figure S6: Selection of hydrolases for the degradation of non-sulfated carrageenan oligosaccharides. (**A**) Production of DA by the action of different exo-(α-1,3)-3,6-anhydro-D-galactosidases: DA production was monitored at 340 nm by the reduction of NAD^+ using CaDADH. 1 % crude κ-carrageenan-oligosaccharides were incubated with CaCgS2, WcBGH, CaDADH, 1 mM NAD^{+}, and the respective exo-(α-1,3)-3,6-anhydro-D-galactosidases at 37 °C in 20 mM Tris-HCl pH 7.5 in a volume of 200 µL. (B) Production of DA from κ-carrageenan with or without the addition of WcBGH.

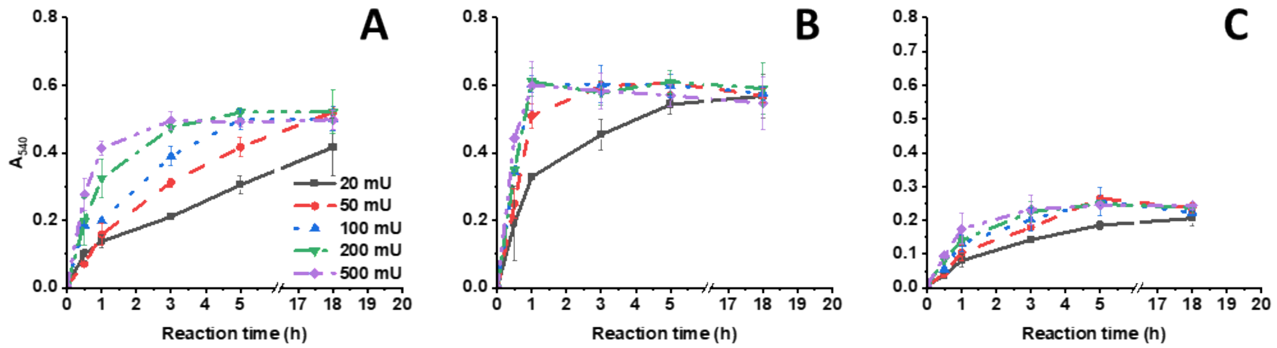

Figure S7: Hydrolysis of 0.5 % solutions of ι-carrageenan (**A**), κ-carrageenan (**B**) and furcellaran (**C**) by the action of BovGH16 over 18 h in 20 mM Tris-HCl pH 7.5 at 37 °C. Production of reducing ends was determined at different time points by DNS-assay.

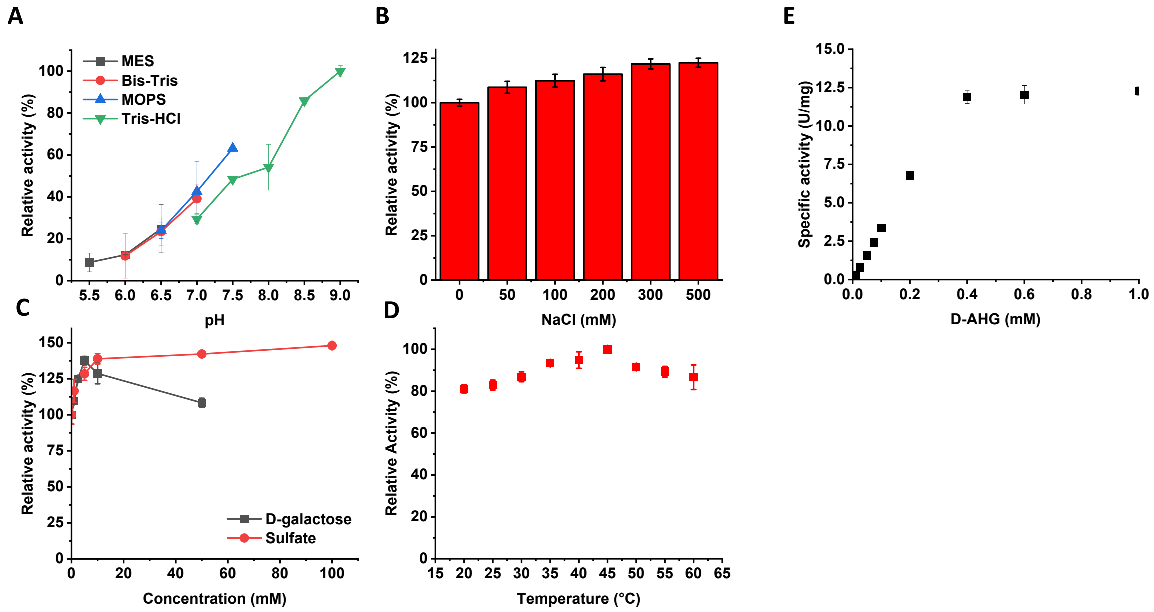

Figure S8: Biochemical characterization of CaDADH. (**A**) pH-dependency of CaDADH-activity in 20 mM of different buffers from pH 5.5 to pH 9. (**B**) Influence of NaCl on CaDADH- activity. (**C**) Influence of D-galactose and sulfate on CaDADH-activity. (**D**) Temperature-dependency of CaDADH-activity. (**E**) Kinetic analysis of CaCADH. With exception of the respective optimization experiments, all experiments were performed with 1 mM NAD^+ and 1 mM DA in 20 mM Tris-HCl pH 7.5 at 37 °C.

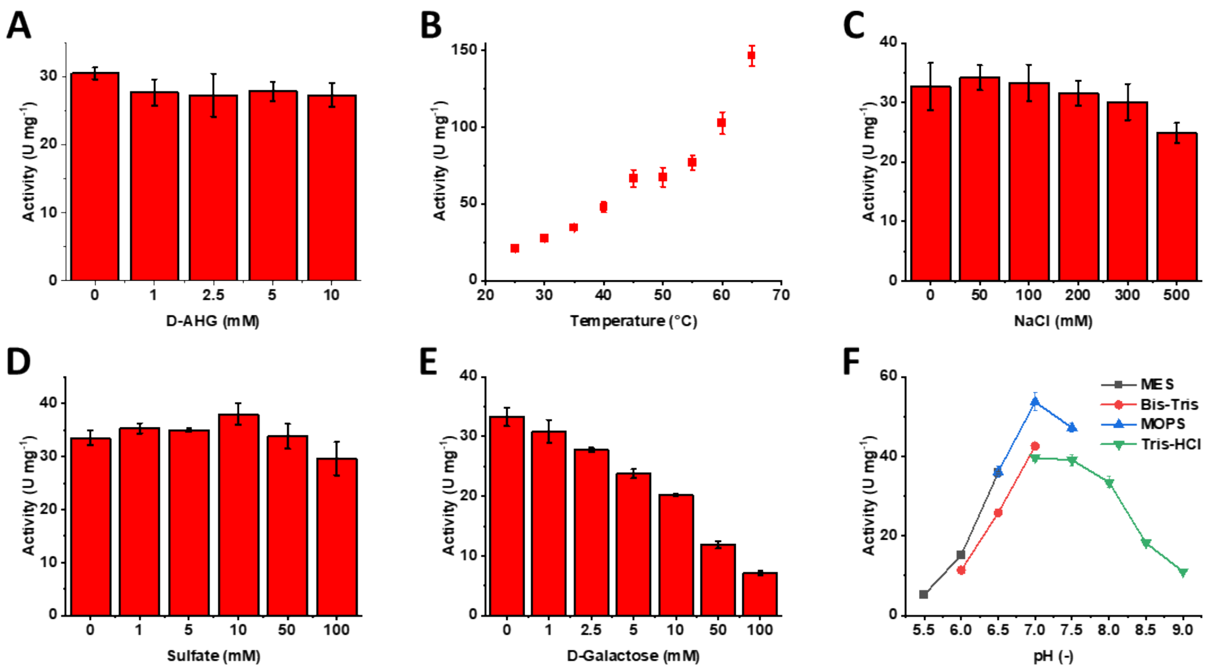

Figure S9: Biochemical Characterization of WcBGH using pNPS as substrate. (**A**) Influence of increasing concentrations of DA on WcBGH activity. (**B**) The activity of WcBGH at different temperatures. (**C**) Influence of NaCl on enzyme activity. (**D**) Influence of increasing amounts of sulfate on WcBGH activity. (**E**) Effect of D-galactose on WcBGH activity (**F**) pH-profile of WcBGH. All experiments were performed in 20 mM Tris-HCl pH 7.5 at 37 °C (with exception of the temperature profile).

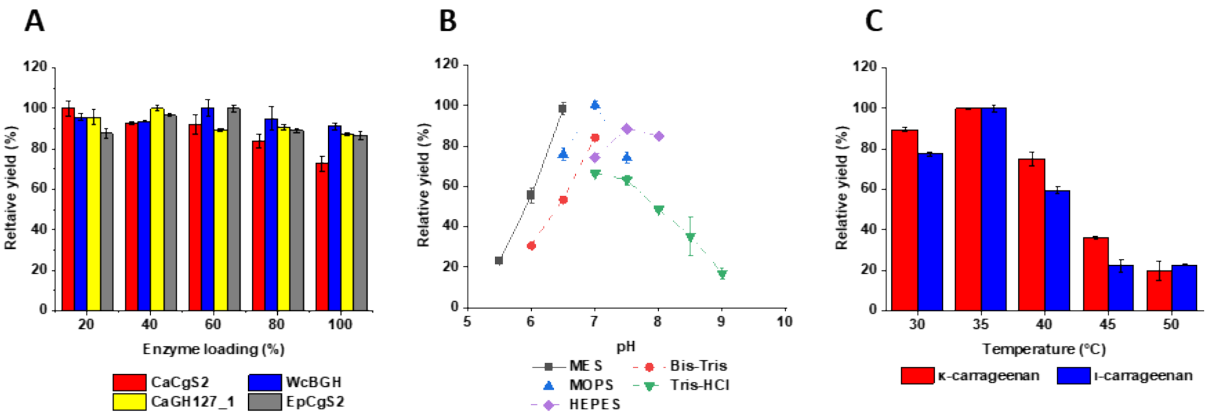

Figure S10: Optimization of cascade performance: (**A**) Testing of different enzyme loadings for the utilization of 0.5 % pre-hydrolyzed κ-carrageenan: 100 % refers to 2.5 mg mL^{-1} CaCgS1, 0.667 mg mL^{-1} EpCgS2, 0,75 mg mL^{-1} CaGH127_1 and 0.166 mg mL^{-1} WcBGH. When a single enzyme loading was examined, the other concentrations were set to 100 %. Experiments were performed at pH 7.5 in 20 mM Tris-HCl at 37 °C for 12 h. (**B**) Performance of the enzyme-ratio optimized cascade at different pH at 20 mM of the indicated buffers. Reactions were performed at 37 °C for 12 h. (**C**) Cascade performance at different temperatures. Reactions were performed in 20 mM Tris-HCl pH 7.5 for 12 h.

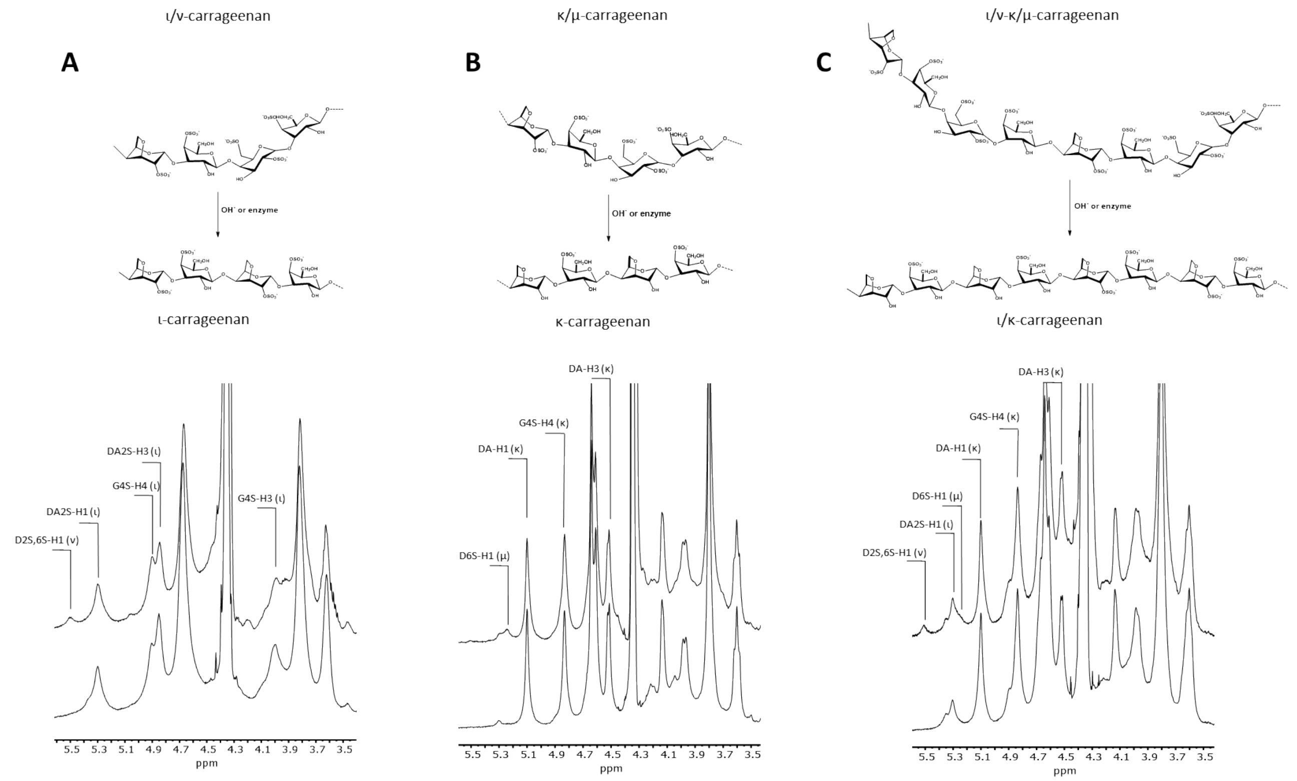

Figure S11: Structures and ^{1}H-NMR spectra of aqueous and alkaline extracted carrageenans from *Eucheuma. spinosum* (**A**), *Kappaphycus alvarezii* (**B**) and *Chondrus Crispus* (**C**). The top spectra show aqueous-extracted carrageenans containing significant amounts of the biosynthetic precursors μ- and ν-carrageenan, which disappear after alkaline extraction (bottom spectra).

Supplementary References

1. Préchoux, A.; Genicot, S.; Rogniaux, H.; Helbert, W., Controlling Carrageenan Structure Using a Novel Formylglycine-Dependent Sulfatase, an Endo-4S-iota-Carrageenan Sulfatase. *Marine Biotechnology* **2013,** *15* (3), 265-274.
2. Préchoux, A.; Genicot, S.; Rogniaux, H.; Helbert, W., Enzyme-Assisted Preparation of Furcellaran-Like κ-/β-Carrageenan. *Marine Biotechnology* **2016,** *18* (1), 133-143.
3. Poulet, L.; Mathieu, S.; Drouillard, S.; Buon, L.; Loiodice, M.; Helbert, W., α-Carrageenan: An alternative route for the heterogenous phase degradation of hybrid ι-/κ-carrageenan. *Algal Research* **2023,** *71*, 103049.
4. Ficko-Blean, E.; Préchoux, A.; Thomas, F.; Rochat, T.; Larocque, R.; Zhu, Y.; Stam, M.; Génicot, S.; Jam, M.; Calteau, A.; Viart, B.; Ropartz, D.; Pérez-Pascual, D.; Correc, G.; Matard-Mann, M.; Stubbs, K. A.; Rogniaux, H.; Jeudy, A.; Barbeyron, T.; Médigue, C.; Czjzek, M.; Vallenet, D.; McBride, M. J.; Duchaud, E.; Michel, G., Carrageenan catabolism is encoded by a complex regulon in marine heterotrophic bacteria. *Nature Communications* **2017,** *8* (1), 1685.
5. Hettle, A. G.; Vickers, C.; Robb, C. S.; Liu, F.; Withers, S. G.; Hehemann, J. H.; Boraston, A. B., The Molecular Basis of Polysaccharide Sulfatase Activity and a Nomenclature for Catalytic Subsites in this Class of Enzyme. *Structure* **2018,** *26* (5), 747-758.e4.
6. Kumar, S.; Stecher, G.; Li, M.; Knyaz, C.; Tamura, K., MEGA X: Molecular Evolutionary Genetics Analysis across Computing Platforms. *Mol Biol Evol* **2018,** *35* (6), 1547-1549.
7. Jones, D. T.; Taylor, W. R.; Thornton, J. M., The rapid generation of mutation data matrices from protein sequences. *Comput Appl Biosci* **1992,** *8* (3), 275-82.
8. Edgar, R. C., MUSCLE: multiple sequence alignment with high accuracy and high throughput. *Nucleic Acids Res* **2004,** *32* (5), 1792-7.
9. Barbeyron, T.; Brillet-Guéguen, L.; Carré, W.; Carrière, C.; Caron, C.; Czjzek, M.; Hoebeke, M.; Michel, G., Matching the Diversity of Sulfated Biomolecules: Creation of a Classification Database for Sulfatases Reflecting Their Substrate Specificity. *PLOS ONE* **2016,** *11* (10), e0164846.
10. van de Velde, F.; Knutsen, S. H.; Usov, A. I.; Rollema, H. S.; Cerezo, A. S., 1H and 13C high resolution NMR spectroscopy of carrageenans: application in research and industry. *Trends in Food Science & Technology* **2002,** *13* (3), 73-92.
